# Maternal adverse childhood experiences (ACEs) and DNA methylation of newborns in cord blood

**DOI:** 10.1186/s13148-023-01581-y

**Published:** 2023-10-16

**Authors:** Phillip Collender, Anne K. Bozack, Stephanie Veazie, Jamaji C. Nwanaji-Enwerem, Lars Van Der Laan, Katherine Kogut, Corinne Riddell, Brenda Eskenazi, Nina Holland, Julianna Deardorff, Andres Cardenas

**Affiliations:** 1 Division of Environmental Health Sciences, University of California, Berkeley, CA USA; 2 Department of Epidemiology and Population Health, Stanford University School of Medicine, Research Park, 1701 Page Mill Road, Stanford, CA 94304 USA; 3 Division of Epidemiology, School of Public Health, University of California, Berkeley, CA USA; 4 Gangarosa Department of Environmental Health, Rollins School of Public Health, Emory University, Atlanta, GA USA; 5 Department of Emergency Medicine, School of Medicine, Emory University, Atlanta, GA USA; 6 Department of Statistics, University of Washington, Seattle, WA USA; 7 Center for Environmental Research of Community Health, CERCH, School of Public Health, University of California, Berkeley, CA USA; 8 Division of Biostatistics, School of Public Health, University of California, Berkeley, CA USA; 9 Division of Community Health Sciences, School of Public Health, University of California, Berkeley, CA USA; 10 Department of Pediatrics, Stanford University School of Medicine, Stanford, CA USA

**Keywords:** ACEs, DNA methylation, Adversity, Epigenetic programming

## Abstract

**Background:**

Adverse childhood experiences (ACEs) increase the risk of poor health outcomes later in life. Psychosocial stressors may also have intergenerational health effects by which parental ACEs are associated with mental and physical health of children. Epigenetic programming may be one mechanism linking parental ACEs to child health. This study aimed to investigate epigenome-wide associations of maternal preconception ACEs with DNA methylation patterns of children. In the Center for the Health Assessment of Mothers and Children of Salinas study, cord blood DNA methylation was measured using the Illumina HumanMethylation450 BeadChip. Preconception ACEs, which occurred during the mothers’ childhoods, were collected using a standard ACE questionnaire including 10 ACE indicators. Maternal ACE exposures were defined in this study as (1) the total number of ACEs; (2) the total number of ACEs categorized as 0, 1–3, and > 4; and (3) individual ACEs. Associations of ACE exposures with differential methylated positions, regions, and CpG modules determined using weighted gene co-expression network analysis were evaluated adjusting for covariates.

**Results:**

Data on maternal ACEs and cord blood DNA methylation were available for 196 mother/newborn pairs. One differential methylated position was associated with maternal experience of emotional abuse (cg05486260/*FAM135B* gene; *q* value < 0.05). Five differential methylated regions were significantly associated with the total number of ACEs, and 36 unique differential methylated regions were associated with individual ACEs (Šidák *p* value < 0.05). Fifteen CpG modules were significantly correlated with the total number of ACEs or individual ACEs, of which 8 remained significant in fully adjusted models (*p* value < 0.05). Significant modules were enriched for pathways related to neurological and immune development and function.

**Conclusions:**

Maternal ACEs prior to conception were associated with cord blood DNA methylation of offspring at birth. Although there was limited overlap between differential methylated regions and CpGs in modules associated with ACE exposures, statistically significant regions and networks were related to genes involved in neurological and immune function. Findings may provide insights to pathways linking psychosocial stressors to health. Further research is needed to understand the relationship between changes in DNA methylation and child health.

**Supplementary Information:**

The online version contains supplementary material available at 10.1186/s13148-023-01581-y.

## Introduction

Adverse childhood experiences (ACEs) encompass a set of potentially traumatic events that occur during childhood, before age 18 years, and are associated with poor health outcomes during adolescence and adulthood [1]. Examples of ACEs include physical and emotional neglect, substance abuse, parental divorce, incarceration of family members, mental illness in the family, and physical, emotional, and sexual abuse. ACEs are a pervasive issue with around 60% of adults in the USA reporting experiencing at least one ACE before age 18, and around 22% reporting experiencing 3 or more ACEs [1, 2]. Of note, ACEs are more prevalent among children in racially and ethnically marginalized populations as well as those residing in low- and middle-income countries [2–4].

ACEs are a significant public health issue because they have negative impacts on mental and physical health, education, and job opportunities. During child development, they, along with other social determinants of health, contribute to toxic stress, which can cause neurodevelopmental disruption, epigenetic changes, and reprogramming of stress and immune regulatory systems that affect a child’s developmental trajectory and life course [5, 6]. Through these mechanisms, they have been associated with increased risk of many negative physical, psychological, and social outcomes during childhood, including asthma, headaches, obesity, attention-deficit/hyperactivity disorder, and earlier use of alcohol [7]. During adulthood, ACEs have been linked to an increased risk of cardiovascular disease, chronic obstructive pulmonary disease, dementia, suicide attempts, and injection drug use [6, 8, 9]. These associations can contribute to billions of dollars of economic and social costs each year; a 10% reduction in ACE prevalence in North America is estimated to result in an annual savings of 1 million disability-adjusted life years or $56 billion [10].

There is growing interest in how adverse events experienced by parents affect health outcomes in their offspring. Maternal preconception ACEs have been associated with increased risk of hypertensive disorders of pregnancy, preterm birth, and prenatal depressive symptoms. Maternal psychological challenges have also been shown to increase child behavioral dysregulation [11]. This evidence demonstrates that psychosocial stressors experienced early in the life of pregnant individuals can have downstream effects on the prenatal and postnatal time periods [12, 13]. The effect of maternal preconception ACEs on infant developmental outcomes can occur through biophysical and behavioral mechanisms related to increased prenatal health risks, pregnancy psychosocial risks, and postpartum psychosocial risks [14]. Children of mothers with a high number of ACEs were found to have increased risk for depressive symptoms, anxiety, aggression, hyperactivity, and temperament issues [15, 16]. The development of negative psychosocial outcomes among children of mothers with ACEs may be attributable to prenatal substance use, exacerbated stress and depression, emotional maladaptive behaviors, and biological programming, such as through epigenetic changes, during the prenatal and postnatal periods [5, 16, 17]. For example, a greater number of maternal ACEs has been associated with increased epigenetic age acceleration among offspring in the current cohort [18], as well as shorter telomere length throughout infancy in other studies, which contributed to higher risk for maladaptive externalizing behaviors at 18 months of age [19]. Studies in animal models have also supported inter- and transgenerational inheritance of trauma through non-genetic inheritance involving the germline [20]. Notably, in mouse models, behavioral alterations have been observed in the F2 generation following chronic stress exposure [21]. In a mechanistic study, trauma experienced by male mice-modified microRNAs of sperm, and injection of sperm microRNAs into fertilized wild-type oocytes conferred trauma-related behavioral changes to offspring [22], providing evidence that intergenerational effects of trauma are due in part to molecular mechanisms independent of parenting behaviors.

Changes in DNA methylation (DNAm) that occur during fetal development and throughout life and offspring inheritance of maternal epigenetic patterns may also confer vulnerability to many pathologic conditions [23]. Associations of cumulative lifetime maternal stress and maternal stress experienced during pregnancy with DNAm of offspring have been studied in epigenome-wide association studies (EWAS) [24, 25], including in a large meta-analysis of 12 cohorts [26]. EWAS of ACEs and DNAm in adulthood [27], as well as EWAS [28, 29] and candidate-gene approaches [30, 31] of maternal preconception ACE exposures and offspring DNAm support the hypothesis that ACEs affect differential DNAm of specific genes. However, these studies have focused on the number of ACEs rather than ACE categories and findings have been inconsistent across studies.

In the Center for the Health Assessment of Mothers and Children of Salinas (CHAMACOS) study, a longitudinal birth cohort from a predominantly Mexican immigrant farmworker population in California [32], we previously reported associations between maternal ACEs and children’s epigenetic age acceleration [18]. In the current study, we expand upon this research by performing an EWAS among CHAMACOS participants to assess the relationship between maternal preconception ACEs, analyzed as total number of ACEs and individual ACEs reported, and newborn cord blood DNAm patterns.

## Results

### Participant characteristics

From an initial cohort of 601 mother/newborn pairs, 372 had high-quality DNAm data. Mothers’ ACEs were collected using an adaptation of the ACE questionnaire [33] including 10 indicators, which was administrated approximately 18 years after the birth of the index child. A total of 203 mother/newborn pairs had data on DNAm and any ACE indicator; missingness was primarily due to loss to follow-up. Our analyses included 196 mother/newborn pairs with full data on maternal ACEs, cord blood DNAm, and other covariates (Additional file 1: Fig. S1). Characteristics of maternal/newborn pairs were similar for those included in analyses (Table 1) and the full sample with available DNAm data (Additional file 1: Table S1).

**Table 1:** Participant characteristics and adverse childhood experiences (ACEs) of mother/newborn pairs included in analyses

Characteristic (*N* = 196)	Mean ± SD or *N* (%)
Maternal age at delivery (years)	25.7 ± 4.8
Maternal parity (births)	1.2 ± 1.1
Maternal pre-pregnancy BMI	27.2 ± 5.1
*Highest level of education attained by mother*	
6th grade or lower	80 (40.8%)
7–12th grade	79 (40.3%)
High school graduate	37 (18.9%)
*Mother ever smoked during pregnancy*	
Yes	8 (4.1%)
No	188 (95.9%)
*Mother’s marital status at time of birth*	
Married	85 (43.4%)
Living as married	76 (38.8%)
Separated	7 (3.6%)
Divorced	3 (1.5%)
Single, never married	25 (12.8%)
*Mother’s country of origin*	
USA	22 (11.2%)
Mexico	172 (87.8%)
Other	2 (1.0%)
*Years mother spent in USA*	
≤ 1 years	34 (17.3%)
2–5 years	53 (27.0%)
6–10 years	57 (29.1%)
11 + years	52 (26.5%)
*Maternal ACEs*	
Emotional abuse	43 (21.9%)
Physical abuse	47 (24.0%)
Sexual abuse	27 (13.8%)
Emotional neglect	44 (22.4%)
Physical neglect	31 (15.8%)
Domestic violence in household	33 (16.8%)
Substance abuse in household	40 (20.4%)
Mental illness in household	19 (9.7%)
Household member incarcerated	17 (8.7%)
Parents divorced	42 (21.4%)
*Total number of ACEs reported*	
0 ACEs	101 (51.5%)
1–3 ACEs	52 (26.5%)
4–10 ACEs	43 (21.9%)
*Newborn sex*	
Male	94 (48.0%)
Female	102 (52.0%)
Gestational age (weeks)	39.1 ± 1.5

The mean ± standard deviation (SD) maternal age at delivery was 25.7 ± 4.8 years (Table 1). On average, mothers had a parity of 1.2 ± 1.1 births and had pre-pregnancy body mass index (BMI) of 27.2 ± 5.1. Forty percent of mothers (*n *= 80; 40.8%) had 6th grade or lower education, while 40.3% (*n *= 79) attained 7th–12th grade education and 18.9% (*n *= 37) graduated from high school. Eight mothers (4.1%) reported ever smoking while pregnant. Forty-three percent of mothers were married (*n *= 85; 43.4%) and 38.8% were living as married (*n *= 76) at the time of delivery, while 5.1% were separated or divorced (*n *= 10), and 12.8% were single and had never married (*n *= 25). The majority of mothers reported Mexico as their country of origin (*n *= 172; 87.8%), and having spent ≤ 1 (*n *= 34; 17.3%), 2–5 (*n *= 53; 27.0%), or 6–10 (*n *= 57; 29.1%) years in the USA. Slightly fewer than half of newborns were male (*n *= 94; 48.0%), and the mean gestational age was 39.1 ± 1.5 weeks.

### Maternal adverse childhood experiences (ACEs)

Approximately half of mothers (*n *= 101; 51.5%) reported no ACEs, while 26.5% (*n *= 52) reported 1–3 ACEs, and 21.9% (*n *= 43) reported 4–10 ACEs (Table 1). The ACEs most frequently reported by mothers were physical abuse (*n *= 47; 24.0%), emotional neglect (*n *= 44; 22.4%), emotional abuse (*n *= 43; 21.9%), parental divorce (*n *= 42; 21.4%), and substance abuse in the household (*n *= 40; 20.4%). The report of any ACE increased the odds of every other ACE, with pairwise odds ratios ranging from 3.4 for domestic violence and physical neglect (95% confidence interval (CI): 1.1, 9.9) to 51.4 for emotional and physical abuse (95% CI: 19.5, 135.4) (Additional file 1: Fig. S2). The maximum variance inflation factor for the mutually adjusted model matrix including all ACEs was 3.30, indicating no major issues with collinearity (data not shown).

### CpG-by-CpG epigenome-wide association study (EWAS) analyses

Associations with individual cytosine-phosphate-guanine (CpG) dinucleotides in cord blood were analyzed using three complementary approaches to defining maternal ACE exposures: (1) total number of ACEs modeled linearly; (2) total number of ACEs categorized as 0, 1–3, or 4–10, allowing for nonlinear effects; and (3) each of the 10 ACEs in a mutually adjusted model. Models were adjusted for a priori selected confounders or precision variables of newborn sex, gestational age, and cord blood estimated cell type proportions, and maternal parity, pre-pregnancy BMI, age at delivery, educational attainment, smoking during pregnancy, and marital status. Within each model, we adjusted for multiple comparisons (i.e., number of CpGs x number of exposure variables) by calculating *q* values using the false discovery rate (FDR) control approach of Storey and Tibshirani [34]. Differentially methylated positions (DMPs) were defined as CpGs with *q* values ≤ 0.05. CpG-by-CpG results from the EWAS are available at the study’s Open Science Framework (OSF) repository at https://osf.io/ync5t/.

We did not identify any DMPs associated with the total number of ACEs modeled linearly (Manhattan plot presented in Additional file 1: Fig. S3) or modeled categorically (Manhattan plots in Additional file 1: Fig. S4). When individual ACEs were included in a mutually adjusted model (Manhattan plots in Additional file 1: Fig. S5), we observed one DMP positively associated with emotional abuse (cg05486260; *FAM135B*) (*q* value < 0.05) (Table 2).

**Table 2:** Differentially methylated position (DMP; *q* value < 0.05) associated with maternal adverse childhood experiences (ACEs)

ACE	CpG	Methyl. OR	*q* value	Baseline maximum likelihood (95% CI) % methylated ^a^	Maximum likelihood (95% CI) % methylation change ^b^	Chr	Position (bp) ^c^	Gene annotation	Feature category
Emotional abuse	cg05486260	1.44	0.01	71.03 (65.74, 75.80)	6.92 (4.82, 9.00)	8	139,206,451	*FAM135B*	Body

^a^Baseline measures were obtained from fitted models by plugging in mean values for continuous variables or most-frequently observed values for categorical variables. Thus the baseline prediction is for a female child with gestational age of 273.36 days, whose mother had 1.19 previous births with gestational age $\ge$ 24 weeks, did not smoke during pregnancy, was married at time of birth, had educational attainment at or below 6th grade, was 25.73 years old at time of birth, and whose cord blood cell type composition was 18.94% CD4 + T cells, 9.12% CD8 + T cells, 0.73% natural killer cells, 18.33% B Cells, 10.92% monocytes, 40.63% granulocytes, 1.33% nucleated red blood cells

^b^Exposed measures were obtained by plugging in the same values used to represent the mean/most frequently observed individual for baseline and setting exposure variables to 1. The Differences between exposed and baseline methylation were Monte Carlo simulated using 3000 draws from a multivariate normal distribution, followed by inverse logit transformation and subtraction

c. hg19 assembly

Genomic inflation was evaluated using Q–Q plots and the genomic inflation factor (λ) (Additional file 1: Figs. S6–S8). In the mutually adjusted model of individual ACEs, some degree of genomic inflation, i.e., observed *p* values systematically lower or more significant than the expected under the null hypothesis distribution, was observed for emotional neglect (*λ *= 1.65), substance abuse (*λ *= 1.54), and mental illness (*λ *= 1.40) (Additional file 1: Fig. S8). Deflation, perhaps corresponding to a lack of statistical power, was evident for emotional abuse (*λ *= 0.86), physical abuse (*λ *= 0.89), and parental divorce (*λ *= 0.67). Deflation was also present for 1–3 versus 0 ACEs in the categorical model of total ACEs (*λ *= 0.75), although deflation was less evident for 4–10 versus 0 ACEs (*λ *= 0.97) or 4–10 versus 1–3 ACEs (*λ *= 1.13) (Additional file 1: Fig. S7).

We looked up the DMP cg05486260 in the EWAS Catalog database [35]. This CpG has previously been associated with gestational age in fetal brain tissue [36] and with age in a longitudinal analysis of cord blood and blood collected in childhood and adolescence [37].

We also evaluated if results from two previous studies replicated in our analyses. Moore et al. investigated associations between the number of maternal ACEs and DNAm measured in blood collected from infants at age 3 months (*N *= 92) [29]. The top CpG located in each of 142 correlated methylated regions (*p *< 0.005) as well as 189 individual CpGs (*p* < 0.0005) were reported, of which 314 were included in our study. Among these CpGs, 129 were nominally significant (*p* value < 0.05) in at least one of our models (total number of ACEs: 14 CpGs; total number of ACEs categorical, 1–3 vs. 0: 8 CpGs; 4–10 vs. 0: 12 CpGs; 4–10 vs. 1–3: 15 CpGs; mutually adjusted, emotional abuse: 13 CpGs; physical abuse: 11 CpGs; sexual abuse: 11 CpGs; emotional neglect: 19 CpGs; physical neglect: 10 CpGs; domestic violence: 15 CpGs; substance abuse: 20 CpGs; mental illness: 23 CpGs; incarceration: 13 CpGs; divorce: 9 CpGs), although none remained significant after adjusting for multiple comparisons in our EWAS (Additional file 2: Table S1).

Kotsakis Ruehlmann et al. conducted a meta-analysis of maternal prenatal stressors in the Pregnancy and Childhood Epigenetics (PACE) consortium (*p* value < 2.4 × 10^–7^) [26]. Of the five DMPs associated with prenatal cumulative stress or individual stress domains in PACE, four were analyzed in our study. Only one DMP achieved nominal significance (*p* value < 0.05) in any of our EWAS (Additional file 2: Table S2). The site, cg14228885, annotated to *APTX*, had higher methylation among mothers who reported experiencing conflict with family and friends during pregnancy in eight cohorts included in the PACE meta-analysis, and had higher methylation among mothers who reported substance abuse in the household in our study (*p* = 0.005).

### Regional epigenome-wide association analyses

Differentially methylated regions (DMRs) in cord blood were identified using comb-p with a Šidák multiple testing correction [38], and significant DMRs were defined as regions with a Šidák *p* value ≤ 0.05. DMRs were associated with the total number of ACEs modeled linearly (4 DMRs), the total number of ACEs modeled categorically (1 DMR for 4–10 ACEs vs. 0 ACEs), and individual ACEs in a mutually adjusted model (3 DMRs for emotional abuse, 6 DMRs for sexual abuse, 2 DMRs for emotional neglect, 9 DMRs for physical neglect, 4 DMRs for domestic violence, 3 DMRs for substance abuse, 5 DMRs for mental illness, 3 DMRs for incarceration, and 3 DMRs for parental divorce) (Šidák *p* value < 0.05) (Table 3).

**Table 3:** Differentially methylated regions (DMRs) associated with maternal adverse childhood experiences (ACEs)

Chr	Start ^a^	End	Width	# CpGs	Šidák *p *value	Mean OR (2.5, 97.5 percentile)	Baseline maximum likelihood (95% Range) % methylated ^b^	Maximum likelihood (95% Range) % methylation change ^c^	Gene annotation		Feature category
*Total number of ACEs*											
6	31,734,265	31,734,450	186	5	$7.14\times {10}^{-3}$	0.98 (0.97, 0.98)	39.42 (23.59, 51.51)	− 0.56 (− 0.72, − 0.40)	*VWA7*		*Body*
7	39,170,545	39,171,071	527	5	$4.82\times {10}^{-8}$	0.94 (0.93, 0.96)	84.94 (72.31, 94.29)	− 0.81 (− 1.53, − 0.22)	*POU6F2*		*Body*
11	316,088	316,505	418	4	$5.72\times {10}^{-10}$	0.95 (0.93, 0.97)	39.69 (17.51, 61.91)	− 1.05 (− 1.41, − 0.66)	*–*		*–*
16	84,346,770	84,346,979	210	4	$3.98\times {10}^{-6}$	1.02 (1.02, 1.03)	63.93 (61.88, 67.33)	0.50 (0.44, 0.54)	*WFDC1*		*Body*
*Total number of ACEs categorized*											
*4–10 versus 0 total ACEs*											
7	73,894,932	73,895,279	348	5	$6.85\times {10}^{-13}$	1.20 (1.11, 1.29)	33.81 (25.07, 42.00)	4.12 (2.02, 6.36)	*GTF2IRD1*		*5’UTR*
*Mutually adjusted ACEs*											
*Emotional abuse*											
3	126,007,325	126,007,564	240	4	$3.26\times {10}^{-7}$	0.69 (0.61, 0.83)	28.89 (22.20, 37.96)	− 7.04 (− 8.49, − 4.36)	–		
8	95,962,132	95,962,512	381	6	$1.36\times {10}^{-6}$	1.41 (1.33, 1.50)	44.33 (31.06, 59.17)	9.48 (7.36, 11.45)	*TP53INP1*		*TSS1500*
15	74,218,577	74,218,970	394	9	$6.76\times {10}^{-10}$	1.23 (1.16, 1.36)	46.63 (34.23, 61.96)	5.12 (3.75, 6.87)	*LOXL1,* *LOXL1-AS1*		*TSS200, 5'UTR, 1st Exon*
*Sexual abuse*											
2	128,453,260	128,453,533	274	4	$1.17\times {10}^{-5}$	0.44 (0.30, 0.54)	26.07 (14.01, 31.99)	− 12.02 (− 14.43, − 9.04)	*–*		*–*
4	4,543,812	4,544,026	215	8	$6.96\times {10}^{-4}$	1.17 (1.10, 1.25)	2.45 (1.18, 5.48)	0.38 (0.25, 0.61)	*STX18-AS1*		*TSS200, TSS1500*
7	27,155,036	27,155,283	248	5	$3.01\times {10}^{-4}$	0.86 (0.83, 0.90)	95.28 (92.40, 98.32)	− 0.65 (− 1.04, − 0.35)	*HOXA3*		*5’UTR, TSS1500*
7	38,350,921	38,351,155	235	4	$1.17\times {10}^{-4}$	0.61 (0.58, 0.65)	13.52 (9.83, 17.25)	− 4.83 (− 5.94, − 3.69)	*–*		*–*
12	2,339,439	2,339,663	225	3	$2.17\times {10}^{-4}$	0.83 (0.77, 0.87)	44.98 (40.00, 51.01)	− 4.70 (− 6.19, − 3.43)	*CACNA1C*		*Body*
20	36,148,505	36,149,456	952	34	$<1.0\times {10}^{-13}$	0.87 (0.79, 0.94)	58.73 (41.48, 75.15)	− 3.41 (− 5.80, − 1.48)	*BLCAP*		*5’UTR, TSS1500, TSS200*
*Emotional neglect*											
6	31,733,847	31,734,148	302	8	$4.07\times {10}^{-5}$	0.82 (0.77, 0.85)	80.46 (56.65, 92.09)	− 2.95 (− 4.92, − 1.53)	*VWA7*		*Body*
17	40,274,703	40,274,741	39	5	$7.38\times {10}^{-4}$	0.86 (0.81, 0.92)	95.38 (93.90, 96.93)	− 0.86 (− 1.33, − 0.39)	*HSPB9, KAT2A*		*TSS200, TSS1500*
*Physical neglect*											
1	234,367,322	234,367,587	266	4	$4.84\times {10}^{-5}$	1.56 (1.50, 1.64)	70.24 (59.59, 80.26)	8.22 (6.97, 9.59)	*SLC35F3*		*Body*
6	28,829,230	28,829,689	460	19	$6.06\times {10}^{-5}$	1.27 (0.98, 1.58)	83.40 (31.61, 96.99)	1.19 (− 0.45, 2.58)	*LINC01623*		*Body*
6	32,063,487	32,064,307	821	26	$<1.0\times {10}^{-13}$	1.51 (1.17, 1.99)	43.51 (12.35, 78.93)	8.48 (3.03, 16.83)	*TNXB*		*Body*
11	6,291,625	6,292,512	888	7	$8.09\times {10}^{-10}$	1.67 (1.42, 2.06)	82.46 (72.74, 86.98)	6.27 (4.12, 10.16)	*CCKBR*		*Body*
11	67,383,425	67,383,863	439	7	$2.06\times {10}^{-5}$	1.43 (1.34, 1.60)	42.56 (29.66, 63.22)	7.94 (6.31, 10.23)	*–*		
11	68,782,024	68,782,260	237	5	$5.73\times {10}^{-6}$	1.72 (1.37, 2.39)	55.43 (39.63, 75.69)	11.27 (5.05, 20.37)	*MRGPRF*		*Body*
13	20,751,257	20,751,994	738	5	$5.23\times {10}^{-12}$	1.46 (1.40, 1.62)	55.70 (36.31, 88.56)	8.56 (3.16, 13.07)	*–*		
15	42,371,635	42,371,935	301	5	$1.44\times {10}^{-3}$	1.35 (1.24, 1.47)	84.80 (72.88, 92.42)	3.24 (1.97, 5.06)	*PLA2G4D*		*Body*
18	44,562,067	44,562,137	71	6	$4.78\times {10}^{-4}$	1.48 (1.32, 1.63)	97.69 (97.08, 98.31)	0.68 (0.65, 0.71)	*KATNAL2, TCEB3B*		*5’UTR, TSS200*
*Domestic violence in household*											
4	4,543,474	4,544,026	553	11	$2.81\times {10}^{-7}$	0.86 (0.83, 0.91)	2.44 (1.19, 5.20)	− 0.33 (− 0.49, − 0.17)	*STX18, STX18-AS1*		*TSS200, TSS1500, Body, 1st Exon, 5'UTR,*
6	31,803,880	31,804,462	583	7	$4.15\times {10}^{-6}$	1.40 (1.30, 1.57)	26.18 (2.22, 63.76)	3.79 (1.11, 6.02)	*SNHG32, SNORD52*		*5’UTR, TSS1500*
11	368,351	368,761	411	12	$1.42\times {10}^{-6}$	1.24 (1.15, 1.34)	31.02 (10.81, 71.04)	3.71 (1.67, 6.52)	*B4GALNT4*		*TSS1500*
12	4,918,848	4,919,139	292	4	$5.34\times {10}^{-5}$	0.63 (0.54, 0.71)	3.83 (2.60, 6.63)	− 1.32 (− 2.09, − 0.74)	*KCNA6*		*5’UTR, 1st Exon*
*Substance abuse in household*											
6	33,245,619	33,245,780	162	10	$4.30\times {10}^{-3}$	1.10 (1.04, 1.15)	54.43 (26.50, 76.76)	2.31 (1.02, 3.66)	*B3GALT4*		*1st Exon*
17	46,018,923	46,019,185	263	6	$4.36\times {10}^{-8}$	1.32 (1.24, 1.42)	2.14 (1.55, 3.21)	0.67 (0.46, 0.83)	*PNPO*		*1st Exon, Body, 5’UTR*
19	35,615,444	35,615,639	196	3	$4.10\times {10}^{-5}$	1.41 (1.31, 1.49)	5.59 (2.33, 10.33)	1.92 (1.04, 2.86)	*LGI4*		*3’UTR*
*Mental illness in household*											
11	67,383,425	67,383,863	439	7	$1.90\times {10}^{-9}$	0.65 (0.55, 0.73)	42.56 (29.66, 63.22)	− 9.21 (− 11.04, − 6.06)	*–*		
15	81,426,525	81,426,670	146	6	$8.92\times {10}^{-3}$	1.75 (1.65, 1.86)	36.18 (27.69, 43.25)	13.57 (11.17, 14.83)	*CFAP161*		*TSS200, 1st Exon, 5’UTR*
18	77,905,391	77,905,800	410	8	$9.10\times {10}^{-11}$	0.67 (0.59, 0.78)	33.49 (22.27, 46.26)	− 7.87 (− 9.87, − 5.23)	*LOC100130522*		*TSS1500, TSS200*
19	38,794,635	38,794,894	260	5	$7.41\times {10}^{-5}$	0.74 (0.63, 0.80)	26.57 (11.62, 54.30)	− 4.79 (− 6.47, − 2.87)	*C19orf33, YIF1B*		*1st Exon, 3’UTR, 5’UTR, TSS200*
19	50,191,390	50,191,732	343	5	$3.80\times {10}^{-5}$	1.24 (1.15, 1.35)	68.29 (50.47, 93.68)	4.39 (0.90, 6.33)	*C19orf76, PRMT1*		*TSS1500, Body, 3’UTR*
*Household member incarcerated*											
6	33,048,086	33,048,968	883	23	$3.77\times {10}^{-13}$	1.56 (1.18, 2.11)	86.34 (68.71, 97.78)	3.85 (0.62, 7.80)	*HLA-DPB1*		*Body*
7	27,170,600	27,171,155	556	12	$2.98\times {10}^{-6}$	0.74 (0.67, 0.82)	29.67 (15.09, 60.04)	− 5.78 (− 7.90, − 4.79)	*HOXA4*		*TSS1500*
17	47,092,026	47,092,273	248	5	$2.79\times {10}^{-6}$	1.42 (1.26, 1.59)	67.00 (59.08, 75.38)	8.25 (4.82, 10.90)	*IGF2BP1*		*Body*
*Parents divorced*											
5	158,531,180	158,531,512	333	4	$3.63\times {10}^{-6}$	0.83 (0.82, 0.84)	40.29 (14.60, 73.47)	− 3.19 (− 3.68, − 2.37)	*–*		*–*
15	101,093,778	101,093,901	124	3	$9.18\times {10}^{-6}$	0.47 (0.44, 0.53)	72.80 (63.78, 84.96)	− 19.57 (− 23.68, − 12.14)	*–*		*–*
20	61,590,799	61,591,067	269	3	$9.70\times {10}^{-6}$	2.90 (2.77, 3.02)	44.52 (41.39, 47.66)	26.98 (26.05, 27.91)	*SLC17A9*		*Body*

Effects of maternal ACEs were modeled linearly or categorically as 0, 1–3, or 4–10 ACEs; effects of individual ACEs were estimated in a mutually adjusted model

^a^hg19 assembly

^b^Baseline measures were obtained from fitted models by plugging in mean values for continuous variables or most-frequently observed values for categorical variables. Thus the baseline prediction is for a female child with gestational age of 273.36 days, whose mother had 1.19 previous births with gestational age $\ge$ 24 weeks, did not smoke during pregnancy, was married at time of birth, had educational attainment at or below 6th grade, was 25.73 years old at time of birth, and whose cord blood cell type composition was 18.94% CD4 + T cells, 9.12% CD8 + T cells, 0.73% natural killer cells, 18.33% B Cells, 10.92% monocytes, 40.63% granulocytes, 1.33% nucleated red blood cells

^c^Exposed measures were obtained by plugging in the same values used to represent the mean/most frequently observed individual for baseline and setting exposure variables to 1. The Differences between exposed and baseline methylation were Monte Carlo simulated using 3000 draws from a multivariate normal distribution, followed by inverse logit transformation and subtraction

There was minimal overlap between DMRs. The total number of ACEs modeled linearly and emotional neglect in the mutually adjusted model were associated with lower methylation of overlapping DMRs on chromosome 6 (*VWA7*), sexual abuse and domestic violence were associated with overlapping DMRs on chromosome 4 (*STX18-AS1*) with opposite directions of association, and physical neglect and mental illness were associated with a DMR on chromosome 11 (intergenic; chr11: 67,383,425–67,383,863) with opposite directions of association.

### Weighted correlation network analysis

**Fig. 1:**
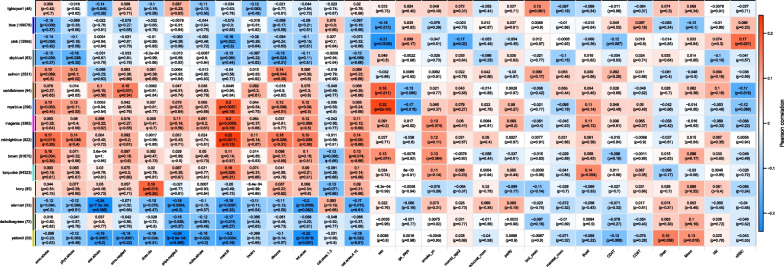
Correlations of module eigengenes (MEs) with maternal adverse childhood experience (ACEs). Pearson correlations with weighted correlation analysis (WGCNA) MEs were calculated for individual maternal ACEs, total maternal ACEs, and covariates. Pearson correlations (*p* values; *q* values) are shown in cells. Only MEs significantly correlated with the total number of ACEs or individual ACEs (*p* < 0.05) are shown

**Fig. 2:**
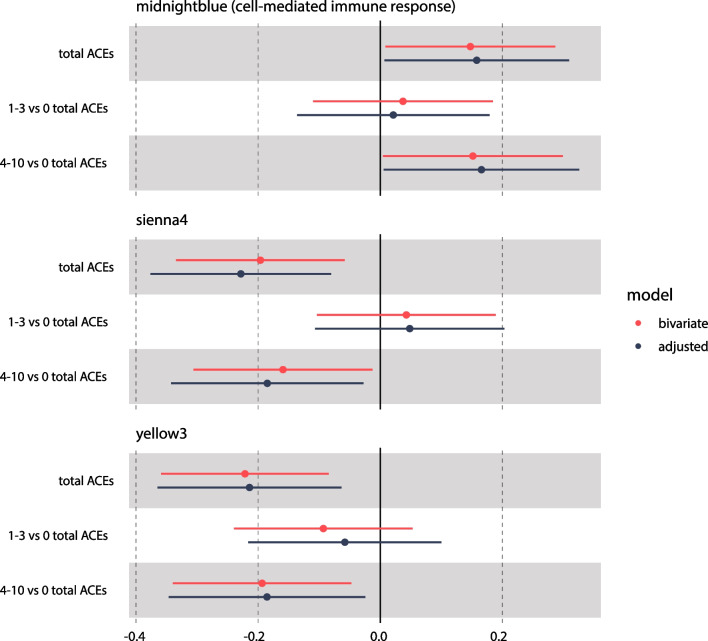
Associations of module eigengenes (MEs) with the number of maternal adverse childhood experiences (ACEs). MEs were derived from weighted correlation analysis (WGCNA). The total number of ACEs was modeled linearly or as a categorical variable of 0, 1–3, or 4–10. Bivariate associations were evaluated using Pearson correlations. Adjusted associations were evaluated using models including newborn sex, gestational age, and cord blood estimated cell type proportions, and maternal parity, pre-pregnancy BMI, age at delivery, educational attainment, smoking during pregnancy, and marital status. Where applicable, summaries of associated biological pathways derived from enrichment analyses are displayed parenthetically next to module names

**Fig. 3:**
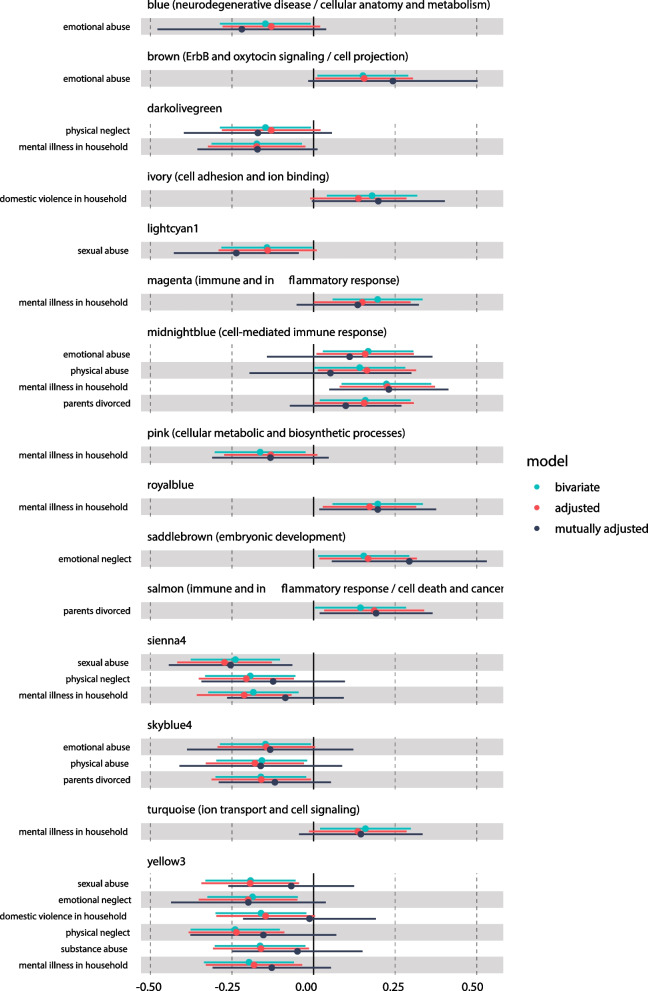
Associations of module eigengenes (MEs) with individual maternal adverse childhood experiences (ACEs). MEs were derived from weighted correlation analysis (WGCNA). Bivariate associations were evaluated using Pearson correlations. Adjusted associations were evaluated using models including newborn sex, gestational age, and cord blood estimated cell type proportions, and maternal parity, pre-pregnancy BMI, age at delivery, educational attainment, smoking during pregnancy, and marital status. Mutually adjusted associations were evaluated using adjusted models including all covariates and ACEs. Where applicable, summaries of associated biological pathways derived from enrichment analyses are displayed parenthetically next to module names

Weighted correlation network analysis, or weighted gene co‐expression network analysis (WGCNA) [39], performed on *M*-values identified 29 modules, ranging in size from 30 CpGs (the minimum module size set in the call to the *blockwiseModules* function) to 109,576 CpGs. One module, which was composed primarily of probes located on the sex chromosomes, was excluded from further analyses. Information required to reconstruct identified WGCNA modules is available at the OSF repository at https://osf.io/ync5t/. Module eigengenes of the 28 remaining modules exhibited strong representativeness, accounting for 41.0–74.0% of the total variation among probes belonging to each module (Additional file 1: Table S2). Median absolute Pearson correlations between included probes and module eigengenes raged from 0.63–0.91. Bivariate associations between module eigengenes (MEs) and the total number of ACEs, individual ACEs, and covariates were evaluated using Pearson correlations (Fig. 1 and Additional file 1: Table S3 and Fig. S9). Modules significantly correlated with ACE exposures (*p* < 0.05) were further analyzed using adjusted models (Figs. 2 and 3 and Additional file 1: Table S3). Pathway enrichment analysis was performed for significant modules using the Kyoto Encyclopedia of Genes and Genomes (KEGG) [40] and Gene Ontology (GO) [41, 42] databases (Table 4 and Additional file 3: Tables S1 and S2).

**Table 4:** Pathways enriched for ACE-associated modules

Module (# CpGs)	KEGG			GO		
	FDR	Pathway	Description	FDR	Pathway	Description
LightCyan1 (48)	–	–	–	–	–	–
Blue (109,576)	7.28 × 10^–10^	hsa05014	Amyotrophic lateral sclerosis	9.79 × 10^–158^	GO:0043231 (CC)	Intracellular membrane-bounded organelle
	9.98 × 10^–09^	hsa05016	Huntington disease	4.50 × 10^–150^	GO:0043229 (CC)	Intracellular organelle
	9.98 × 10^–09^	hsa05022	Pathways of neurodegeneration—multiple diseases	2.35 × 10^–138^	GO:0005622 (CC)	Intracellular anatomical structure
	1.20 × 10^–07^	hsa03040	Spliceosome	9.12 × 10^–120^	GO:0005654 (CC)	Nucleoplasm
	1.20 × 10^–07^	hsa04144	Endocytosis	2.50 × 10^–116^	GO:0043227 (CC)	Membrane-bounded organelle
	1.20 × 10^–07^	hsa04390	Hippo signaling pathway	5.08 × 10^–114^	GO:0043226 (CC)	Organelle
	1.46 × 10^–07^	hsa03010	Ribosome	7.24 × 10^–102^	GO:0005634 (CC)	Nucleus
	2.96 × 10^–07^	hsa05165	Human papillomavirus infection	3.06 × 10^–100^	GO:0044260 (BP)	Cellular macromolecule metabolic process
	5.62 × 10^–07^	hsa05168	Herpes simplex virus 1 infection	4.11 × 10^–90^	GO:0044237 (BP)	Cellular metabolic process
	1.65 × 10^–06^	hsa04110	Cell cycle	2.83 × 10^–89^	GO:0005515 (MF)	Protein binding
Pink (12,955)	0.028	hsa05020	Prion disease	4.95 × 10^–23^	GO:0005654 (CC)	Nucleoplasm
	0.040	hsa03010	Ribosome	3.96 × 10^–19^	GO:0003676 (MF)	Nucleic acid binding
	0.040	hsa03050	Proteasome	5.67 × 10^–19^	GO:0034641 (BP)	Cellular nitrogen compound metabolic process
				1.52 × 10^–18^	GO:0006139 (BP)	Nucleobase-containing compound metabolic process
				1.52 × 10^–18^	GO:0044271 (BP)	Cellular nitrogen compound biosynthetic process
				2.30 × 10^–18^	GO:0070013 (CC)	Intracellular organelle lumen
				2.71 × 10^–18^	GO:0031981 (CC)	Nuclear lumen
				2.71 × 10^–18^	GO:0034645 (BP)	Cellular macromolecule biosynthetic process
				5.37 × 10^–18^	GO:0009059 (BP)	Macromolecule biosynthetic process
				5.37 × 10^–18^	GO:0031974 (CC)	Membrane-enclosed lumen
SkyBlue4 (63)	–	–	–	–	–	–
Salmon (2531)	1.03 × 10^–7^	hsa04659	Th17 cell differentiation	3.75 × 10^–22^	GO:0001775 (BP)	Lymphocyte activation
	2.14 × 10^–7^	hsa04060	Cytokine–cytokine receptor interaction	3.75 × 10^–22^	GO:0046649 (BP)	T cell activation
	1.01 × 10^–6^	hsa04660	T cell receptor signaling pathway	5.89 × 10^–22^	GO:0042110 (BP)	Leukocyte activation
	2.21 × 10^–5^	hsa04658	Th1 and Th2 cell differentiation	7.57 × 10^–22^	GO:0045321 (BP)	Immune system process
	5.19 × 10^–5^	hsa04640	Hematopoietic cell lineage	8.86 × 10^–21^	GO:0002376 (BP)	Immune response
	1.4 × 10^–4^	hsa05166	Human T-cell leukemia virus 1 infection	8.06 × 10^–19^	GO:0006955 (BP)	adaptive immune response
	1.4 × 10^–4^	hsa05235	PD-L1 expression and PD-1 checkpoint pathway in cancer	2.33 × 10^–15^	GO:0002250 (BP)	T cell differentiation
	0.001	hsa04061	Viral protein interaction with cytokine and cytokine receptor	2.63 × 10^–13^	GO:0030217 (BP)	Immune effector process
	0.003	hsa04064	NF-kappa B signaling pathway	3.64 × 10^–13^	GO:0002252 (BP)	Regulation of immune system process
	0.003	hsa05170	Human immunodeficiency virus 1 infection	1.49 × 10^–12^	GO:0002682 (BP)	Lymphocyte differentiation
SaddleBrown (94)	–	–	–	0.003	GO:0048704 (BP)	Embryonic skeletal system morphogenesis
				0.003	GO:0048706 (BP)	Embryonic skeletal system development
				0.007	GO:0009952 (BP)	Anterior/posterior pattern specification
				0.011	GO:0048705 (BP)	Skeletal system morphogenesis
				0.020	GO:0048652 (BP)	Embryonic organ morphogenesis
				0.024	GO:0003002 (BP)	Regionalization
				0.047	GO:0007389 (BP)	Pattern specification process
				0.047	GO:0048568 (BP)	Embryonic organ development
RoyalBlue (296)	–	–	–	–	–	–
Magenta (3363)	–	–	–	7.45 × 10^–6^	GO:0002376 (BP)	Immune system process
				4.76 × 10^–5^	GO:0006955 (BP)	Immune response
				0.004	GO:0006954 (BP)	Inflammatory response
				0.006	GO:0001775 (BP)	Cell activation
				0.03	GO:0002366 (BP)	Leukocyte activation involved in immune response
				0.03	GO:0006887 (BP)	Exocytosis
				0.03	GO:0030097 (BP)	Hemopoiesis
				0.03	GO:0045055 (BP)	Regulated exocytosis
				0.03	GO:0048534 (BP)	Hematopoietic or lymphoid organ development
				0.032	GO:0045321 (BP)	Leukocyte activation
MidnightBlue (822)	1.69 × 10^–7^	hsa04650	Natural killer cell-mediated cytotoxicity	0.008	GO:0002682 (BP)	Regulation of immune system process
	0.016	hsa04660	T cell receptor signaling pathway	0.013	GO:0035556 (BP)	Intracellular signal transduction
	0.016	hsa05135	Yersinia infection	0.019	GO:0006955 (BP)	Immune response
	0.040	hsa05332	Graft-versus-host disease	0.022	GO:0002228 (BP)	Natural killer cell-mediated immunity
				0.033	GO:0050776 (BP)	Regulation of immune response
				0.038	GO:0001906 (BP)	Cell killing
				0.038	GO:0006952 (BP)	Defense response
				0.038	GO:0007159 (BP)	Leukocyte cell–cell adhesion
				0.039	GO:0006909 (BP)	Phagocytosis
				0.039	GO:0046649 (BP)	Lymphocyte activation
Brown (91,675)	0.032	hsa04012	ErbB signaling pathway	0.002	GO:0042995 (CC)	Cell projection
	0.032	hsa04921	Oxytocin signaling pathway	0.002	GO:0043167 (MF)	Ion binding
				0.009	GO:0120025 (CC)	Plasma membrane-bounded cell projection
				0.030	GO:0046873 (MF)	Metal ion transmembrane transporter activity
Turquoise (84,323)	0.001	hsa04020	Calcium signaling pathway	9.94 × 10^–42^	GO:0071944 (CC)	Cell periphery
	0.001	hsa04072	Phospholipase D signaling pathway	2.58 × 10^–32^	GO:0005886 (CC)	Plasma membrane
	0.001	hsa05150	Staphylococcus aureus infection	3.41 × 10^–19^	GO:0031224 (CC)	Intrinsic component of membrane
	0.001	hsa05414	Dilated cardiomyopathy	1.06 × 10^–15^	GO:0016021 (CC)	Integral component of membrane
	0.003	hsa05410	Hypertrophic cardiomyopathy	6.08 × 10^–14^	GO:0005261 (MF)	Cation channel activity
	0.007	hsa04060	Cytokine–cytokine receptor interaction	1.70 × 10^–13^	GO:0046873 (MF)	Metal ion transmembrane transporter activity
	0.007	hsa04080	Neuroactive ligand-receptor interaction	2.07 × 10^–13^	GO:0015267 (MF)	Channel activity
	0.007	hsa04261	Adrenergic signaling in cardiomyocytes	3.39 × 10^–13^	GO:0022803 (MF)	Passive transmembrane transporter activity
	0.007	hsa04512	ECM-receptor interaction	5.09 × 10^–13^	GO:0031226 (CC)	Intrinsic component of plasma membrane
	0.007	hsa04975	Fat digestion and absorption	3.09 × 10^–12^	GO:0005216 (MF)	Ion channel activity
Ivory (85)	–	–	–	5.91 × 10^–28^	GO:0007156 (BP)	Homophilic cell adhesion via plasma membrane adhesion molecules
				2.00 × 10^–24^	GO:0098742 (BP)	Cell–cell adhesion via plasma-membrane adhesion molecules
				1.50 × 10^–17^	GO:0005509 (MF)	Calcium ion binding
				5.37 × 10^–16^	GO:0098609 (BP)	Cell–cell adhesion
				2.27 × 10^–12^	GO:0007155 (BP)	Cell adhesion
				2.27 × 10^–12^	GO:0022610 (BP)	Biological adhesion
				1.13 × 10^–11^	GO:0005887 (CC)	Integral component of plasma membrane
				2.18 × 10^–11^	GO:0031226 (CC)	Intrinsic component of plasma membrane
				3.51 × 10^–04^	GO:0046872 (MF)	Metal ion binding
				4.25 × 10^–04^	GO:0043169 (MF)	Cation binding
Sienna4 (33)	–	–	–	–	–	–
DarkOliveGreen (72)	–	–	–	–	–	–
Yellow3 (59)	–	–	–	–	–	–

The top Kyoto Encyclopedia of Genes and Genomes (KEGG) and Gene Ontology (GO) pathways enriched for modules associated with ACE exposures are listed (FDR < 0.05; up to 10 listed). Pathway analysis performed for modules with eigengenes statistically with significant bivariate correlations (*p* < 0.05) with the total number of ACEs or individual ACE indicators

Fifteen MEs exhibited significant correlations (*p* < 0.05) with the total number of ACEs or individual ACEs (Fig. 1 and Additional file 1: Table S3). Examining relationships between covariates and ACE-associated MEs, we found the greatest correlations between newborn sex and the Blue, Pink, SaddleBrown, and RoyalBlue modules (|*ρ*| range: 0.18–0.22; *p* < 0.05) (Fig. 1). At least one ACE-associated ME was also significantly but weakly correlated with gestational age, maternal age at delivery, % granulocytes, and % nucleated red blood cells (|*ρ*| range: 0.15–0.17; *p* < 0.05). MEs not associated with ACEs were significantly correlated with newborn sex, maternal smoking and age at delivery, and % CD4 + T cells (|*ρ*| range: 0.14–0.22; *p* < 0.05) (Additional file 1: Fig. S9). This suggests that modules represent biologically meaningful DNAm networks, but also that maternal ACE exposures are associated with DNAm networks unique from other biological or sociodemographic factors.

The total number of ACEs modeled linearly was significantly correlated with the MidnightBlue (enriched for cell-mediated immune response; $\rho \hspace{0.17em}$= 0.15; *p* = 0.039), Sienna4 ($\rho \hspace{0.17em}$= − 0.20; *p* = 0.006), and Yellow3 ($\rho \hspace{0.17em}$= − 0.22; *p* = 0.002) MEs (Fig. 1 and Additional file 1: Table S3). After adjustment for covariates, associations with the total number of ACEs remained similar (Fig. 2 and Additional file 1: Table S3). In bivariate and covariate adjusted analyses of the total number of ACEs modeled categorically, 4–10 ACEs versus 0 ACEs was significantly associated with the MidnightBlue, Sienna4, and Yellow3 MEs (*p* < 0.05), consistent with modeling the total number of ACEs linearly. However, estimates of FDRs of linear and categorical associations of total number of ACEs and MEs (*q* values) were > 0.05 (Fig. 1 and Additional file 1: Table S3 and Fig. S9).

In bivariate analyses, mental illness was significantly associated with the most MEs (*p* < 0.05) (8 MEs: Pink, enriched for cellular metabolic and biosynthetic processes; RoyalBlue; Magenta, enriched for immune and inflammatory response; MidnightBlue, enriched for cell-mediated immune response; Turquoise, enriched for ion transport and cell signaling; Sienna4; DarkOliveGreen; and Yellow3) (Fig. 1 and Additional file 1: Table S3). Significant bivariate associations were also observed for emotional abuse (4 MEs: Blue, enriched for neurodegenerative disease/cellular anatomy and metabolism; SkyBlue4; MidnightBlue; and Brown, enriched for ErbB and oxytocin signaling/cell projection), sexual abuse (3 MEs: LightCyan, Sienna4, and Yellow3), divorce (3 MEs: SkyBlue4; Salmon, enriched for immune and inflammatory response/cell death and cancer; and MidnightBlue), physical neglect (3 MEs: Sienna4, DarkOliveGreen, and Yellow3), physical abuse (2 MEs: SkyBlue4 and MidnightBlue), emotional neglect (2 MEs: SaddleBrown, enriched for embryonic development; and Yellow3), domestic violence (2 MEs: Ivory, enriched for cell adhesion and ion binding; and Yellow3), and substance abuse (1 ME: Yellow3). Overall, associations were robust after adjustment for covariates (Fig. 3 and Additional file 1: Table S3). In mutually adjusted models including all ACEs and covariates, the direction of associations was consistent with bivariate analyses, but most associations were nullified. Only associations of mental illness with the RoyalBlue and MidnightBlue MEs, sexual abuse with the LightCyan1 and Sienna4 MEs, divorce with the Salmon ME, and emotional neglect with the SaddleBrown ME remained significant (*p* < 0.05). As with analyses of total number of ACEs, estimated *q* values of bivariate associations between individual ACEs and MEs were > 0.05 (Fig. 1 and Additional file 1: Table S3 and Fig. S9), suggesting caution in interpreting findings.

There was minimal overlap between DMPs, DMRs, and CpGs included in modules associated with ACE exposures. The Blue module (enriched for neurodegenerative disease/cellular anatomy and metabolism) was associated with maternal experiences of emotional abuse and included CpGs in DMRs associated with maternal experiences of sexual abuse, physical neglect, and domestic violence. The Brown module (enriched for ErbB and oxytocin signaling/cell projection) was associated with maternal experience of emotional abuse and included CpGs in DMRs associated with sexual abuse, emotional neglect, and physical neglect. In addition, the Turquoise module (enriched for ion transport and cell signaling), associated with maternal experience of mental illness in the household, included the DMP associated with emotional-abuse (cg0548620, *FAM135B*), as well as CpGs in DMRs associated with physical neglect and domestic violence.

## Discussion

The aim of this study was to examine associations between maternal ACEs and offspring DNAm profiles at birth, including differentially methylated CpGs (DMPs), regions (DMRs), and CpG networks, among mother/newborn pairs who were primarily of Mexican origin and low socioeconomic status [32]. While previous studies have found that maternal adverse experiences are associated with differential methylation of candidate genes among children [30, 31] and accelerated epigenetic aging in children from the current cohort [18], research on the relationship between maternal ACEs and differential methylation on an epigenome-wide level in offspring is nascent. This research may help to elucidate potential biological pathways through which a mother’s adverse physical and psychological experiences impact their child’s health, which can assist in the development of more targeted prevention and therapies. To our knowledge, this is one of the first studies to use cord blood samples to examine the impact of maternal ACEs, including both the number of ACEs reported and individual ACEs, on offspring’s epigenome at large. Using cord blood samples allows us to better understand the impact of maternal adversity on DNAm during fetal development, a time of epigenetic reprogramming with potential downstream consequences for health later in life [43].

We found that emotional abuse experienced by mothers was significantly associated with one DMP in cord blood, cg05486260, annotated to the *FAM135B* gene. In agreement with a study of maternal ACEs and DNAm measured in infant blood and buccal epithelial cells [29] and a study of cord blood [28], we did not find significant associations between the number of maternal ACEs and DMPs after adjustment for multiple comparisons. A recent EWAS of total paternal ACEs and blood collected from infants also found few DMPs, with 8 DMPs in meeting the criteria of *FDR* < 0.2 and a biological threshold |Δ*β *< 0.03|, of which one was significant at FDR < 0.05 [44]. A PACE consortium meta-analysis found 5 DMPs associated with maternal stressors during pregnancy (*p* < 2.4 × 10^–7^) [26], although in our study only one of these CpGs was associated with substance abused in the household at a nominal *p* value < 0.05. Although our findings contribute to a growing body of research linking parental stressful or adverse experiences to epigenetic modifications in children, replication of findings across multiple cohorts is necessary to fully establish and understand this relationship. In our look-up approach, we found limited consistency with results from previous studies [26, 29], which might be due to differences in the populations studied and the specificity, intensity, chronicity, and timing of the exposure.

We found a greater number of associations with DMRs, including significant associations with the total number of ACEs and with emotional abuse, sexual abuse, emotional neglect, physical neglect, domestic violence, substance abuse, mental illness, incarceration, and parent divorce, although there was minimal overlap between the DMRs identified through different analytic models. Previous research has also shown weak associations between total maternal ACEs and correlated methylated regions of infant blood and buccal cells [29]. We also evaluated associations between modules of CpGs, defined using WGCNA, and maternal ACE exposures. WGCNA defines modules of co-methylated CpGs that may have related biological function and therefore, may be advantageous to understanding relationships between DNAm and complex exposures or phenotypes. We found significant associations between the total number of maternal ACEs or individual ACEs and 15 module eigengenes (MEs), which were largely robust after adjusting for covariates (*p* < 0.05). However, *q* values, i.e., estimates of FDRs of accepting any bivariate associations of ACE exposures and MEs, exceeded 0.05, suggesting caution in interpreting findings.

Downstream effects of adversity experienced by parents in early life include depression, anxiety, post-traumatic stress disorder (PTSD), developmental delay, and behavioral problems in their offspring [6, 45]. Moreover, mouse models have demonstrated that maternal trauma during pregnancy is associated with differential expression in offspring brains of genes associated with depression in humans [46]. In the current analysis, we found that maternal report of substance abuse in her household during childhood was associated with offspring regional DNAm of *PNPO*, a protein coding gene involved in the biosynthesis of vitamin B_6_, which is a co-factor for neurotransmitter synthesis. PNPO deficiency has been linked to neurodevelopmental impairment and epilepsy [47]. We also found that maternal childhood experience of sexual abuse was associated with offspring regional methylation of *CACNA1C*. The *CACNA1C* gene encodes for a transmembrane calcium channel subunit. Genetic variation of *CACNA1C* has been associated with neurodevelopmental delays and disorders including schizophrenia, autism, and bipolar disorder [48–51], and DNAm methylation of *CACNA1C* has been associated with bipolar disorder [52], suggesting that maternal ACEs may put children at greater risk for these disorders. Moreover, a cross-sectional study of young adults found a gene × environment interaction in the association of *CACNA1C* polymorphisms with bipolar disorder, with variant carriers exposed to childhood trauma at greater risk [53]. In addition, we found that maternal report of mental illness in the household was significantly associated with the Turquoise co-methylation module, enriched for the neuroactive ligand-receptor interaction and calcium signaling pathways, and that domestic violence in the household was associated with the Ivory co-methylation module, enriched for calcium ion binding. These associations suggest a relationship between preconception maternal ACEs and neurodevelopment and neuronal function in children [54].

Dysregulation of the immune system may be another pathway through which adverse experiences impact health [5, 55, 56]. Stress and adversity in early life has been associated with increased inflammatory markers and susceptibility to infectious diseases. We identified a DMR annotated to the *HLA-DPB1* gene, a member of the major histocompatibility complex (MHC). DNAm of *HLA-DPB1* has previously been associated with PTSD [57, 58]. In our analyses of co-methylation modules, we found several MEs associated with maternal ACE exposures to be enriched for pathways related to immune function. In particular, the Salmon module, associated with parental divorce, and the Magenta module, associated with mental illness in the household, were enriched for immune and inflammatory response pathways. Future studies on ACEs should consider testing co-methylation modules in addition to epigenome-wide CpG testing, which might allow for potential replication of biological pathways affected.

Strengths of this study include use of a validated, standardized measures of maternal ACEs, application of an epigenome-wide approach, and multiple modeling strategies. Our study was also strengthened by the use of cord blood samples which allows us to rule out the possibility that associations are due to similar social and environmental exposures between mothers and children after birth, although we cannot rule out possibility of shared exposures while children were in utero. We also employed multiple analytical techniques which allowed us to investigate associations between ACEs and individual methylation loci, differentially methylated regions, and networks of co-methylated CpGs, in addition to analyzing the total number of ACEs linearly, categorized number of ACEs for nonlinearity, and analyzing associations with individual ACEs mutually adjusting for all other ACEs. Although use of multiple models may increase the risk of type-one errors, we adjusted for multiple comparisons within each model and evaluated statistical significance using a false discovery rate approach (i.e., *q* value ≤ 0.05). However, we chose not to correct for multiple comparisons between models as hypotheses were related.

A limitation of this study is that 176 of the 372 mother/newborn pairs with cord blood DNAm from the original cohort were excluded due to missing data on maternal ACEs. Maternal ACEs were collected at the child’s 18-year follow-up visit, and, therefore, loss to follow-up was a major factor in the missingness of these data. However, characteristics of mother/newborn pairs included in analyses were similar to all pairs with DNAm data, and we do not expect bias due to loss to follow-up. Low response rates to questions about ACEs are also common [59] and may reflect participant hesitation to disclose traumatic or stigmatizing events to researchers. However, in CHAMACOS, mothers were encouraged to respond to ACE questions privately and independently if possible. Five percent of women from the original cohort declined to answer any ACEs questions, and an additional 4% declined to answer between 1 and 9 specific ACEs. Another limitation of this study is there may have been some recall inaccuracy and bias of ACEs exposure, given mothers completed this questionnaire at the 18-year follow-up visit, although retrospective report by adults of serious adverse experiences during childhood is expected to be valid [60].

Our small sample size decreased our power to detect associations with small effect sizes, particularly in CpG-by-CpG analyses, although our sample size was larger than that in previous studies of maternal ACEs and offspring DNAm of candidate genes [30, 31]. It should be noted, however, that a previous EWAS of maternal ACEs and cord blood DNAm in ARIES had a larger sample size (*N *= 896) [28] while a study of maternal ACEs and correlated methylated regions and infant DNAm in APrON had a smaller sample size (*N *= 92 and *N *= 124 for blood and buccal epithelial cells, respectively) [29]. We chose to test for DMRs using comb-p, which has greater power to detect DMRs with small effect sizes compared to alternate regional approaches [61]. However, it should be noted that comb-p may also have a greater Type I error rate [62], and therefore, our findings should be interpreted as candidate regions that may be investigated in larger studies. Due to the small sample size, we were also unable to test for effect modification, such as differential effects by newborn sex, which may influence prenatal programming in response to maternal stress and adverse experiences [28, 63, 64]. In addition, we adjusted models for potential confounders and precision variables selected a priori, including newborn sex and gestational age, cord blood estimated cell type proportions, and maternal pre-pregnancy BMI, age at delivery, parity, educational attainment, marital status, and smoking during pregnancy. These covariates have well-established associations with cord blood DNAm [65–74]. Although these some of these variables follow the experience of preconception ACEs and may be involved in mediating associations between maternal ACEs and offspring DNAm, our findings represent the direct effects of ACEs not acting through those pathways.

Future research should examine the impact of maternal ACEs on children’s DNAm profiles at different points throughout the life course (e.g., adolescence) to evaluate persistence and their role in the sequelae of health outcomes related to ACEs. While our study demonstrates that changes in children’s DNAm possibly reflect maternal adverse experiences prior to conception and birth, prospective, longitudinal cohort studies with repeated measurements may provide opportunities to study how DNAm changes in response to parental ACEs throughout an individual’s life into adulthood. Studies of the impact of maternal ACEs on offspring health or biomarkers in childhood and adolescence must also consider the contribution of the postnatal environment, which may play an important role as a mechanistic pathway or mediator beyond prenatal biological factors. Specifically, maternal anxiety, depression and parenting behaviors have been identified as mediators linking maternal ACEs with children’s internalizing and externalizing symptoms [75–78]. In addition, children’s own direct experience of ACEs must be considered. Future studies should also explore the effects of maternal ACEs on maternal DNAm during pregnancy and at delivery, and to what extent DMPs and DMRs serve as mediators between maternal ACEs and risk of diseases in offspring. These types of studies can help distinguish between environmental and biological pathways between maternal ACEs and childhood health, a key step in developing effective interventions. A recent systematic review [79] also highlighted the potential to investigate the impact of psychosocial interventions on changes in offspring DNAm, which may persist into adulthood [80]. This is a promising strategy for establishing DNAm as a mechanism or biomarker involved in pediatric health, as well as in important method for evaluating the effectiveness of preventive interventions. Finally, because replication of results across cohorts and studies remains a challenge due to small sample sizes and the need to adjust for multiple comparisons [27, 79], including questions on parental and child ACEs in large epigenomic databases may help researchers to conduct larger studies.

## Conclusions

In summary, this study provides further evidence that mothers’ preconception adverse experiences may impact their child’s epigenome at birth. Both gene annotations of DMRs and enrichment analysis of ACE-associated co-methylated modules suggest changes in DNAm related to neurological and immune development and function, pathways previously implicated in response to stressful and adverse experiences. However, larger studies with more diverse populations are needed to fully understand the impact of maternal ACEs on epigenetic markers in children. In addition, further research is needed to investigate how changes in offspring DNAm at birth related to maternal ACEs may affect health later in life.

## Methods

### Study population

From October 1999 through October 2000, 601 pregnant women with $\le$ 20 weeks gestation were recruited into the CHAMACOS study from a predominantly farmworker population of Mexican origin in California’s Salinas Valley [32]. Of these, 527 enrollees had a liveborn singleton delivery in 2000–2001. Enrollees were required to be either English- or Spanish-speaking, eligible for MediCal, planning to deliver at the county hospital, and attending prenatal care visits at one of six local clinics, primarily serving farmworker families. Cord blood samples were collected from 403 newborns for epigenomic analysis. Maternal and child characteristics were retrieved from medical records abstracted by a registered nurse and from interviews conducted by bilingual bicultural well-trained interviewers during the 1st and 2nd trimesters of pregnancy, shortly after delivery, and in regular follow-up interviews conducted most recently when the child was 18 years old. Mothers provided written consent for all study activities. Child written consent was obtained at the 18-year visit. Study activities were approved by the University of California, Berkeley Committee for the Protection of Human Subjects.

### Maternal characteristics

Maternal characteristics included maternal age at delivery, pre-pregnancy BMI, whether the mother smoked at any time during pregnancy, marital status at first interview (married, living as married, separated, divorced, single), educational attainment (≤ 6th grade, 7–12th grade, ≥ 12th grade), and maternal parity.

### Maternal adverse childhood experiences (ACEs)

Data collection of mothers’ ACEs has previously been described [18]. Of the 527 mothers followed to delivery, *N *= 319 (61%) participated in the 18-year follow-up interviews during which data on ACEs were collected using an adaptation of the ACEs questionnaire [33] administered in English or translated to Spanish. The interview was administered primarily aloud due to limited literacy among participants, with a study interviewer reading questions and response options from a computerized questionnaire and keying in the participant’s responses. Participants were encouraged to respond to the ACEs questions privately and independently if possible, though most chose to answer aloud. The questionnaire included 10 self-reported indicators of emotional, physical, or sexual abuse; emotional or physical neglect; substance abuse; incarceration; mental illness or domestic violence in the home; or parental divorce. Participants could decline to respond to one or more ACEs. Partial data on ACEs, i.e., response to one at least one individual ACE, were available for 303 mothers, and complete data used in the current analyses, i.e., responses to all 10 ACEs, were available for 289 mothers. ACEs were categorized as reported (1) or not reported (0).

### Child characteristics at birth

Where possible, gestational age from medical records was calculated based on the self-reported last menstrual period of mothers. In cases where this information was missing or resulted in implausible estimates, ultrasound records were used to estimate gestational age at a weekly resolution. Data on the sex of the children were obtained from physical exams.

### Child DNA methylation data

Cord blood specimens were collected at the time of delivery. DNA was extracted from banked non-heparinized umbilical cord using QIAamp DNA Blood Maxi Kits (Qiagen, Valencia, CA) following a previously described modified version of the manufacturer’s protocol [81]. Aliquots of 1 µg DNA extract were bisulfite converted using Zymo Bisulfite Conversion Kits (Zymo Research, Orange, CA), followed by whole-genome amplification, enzymatic fragmentation, and purification. DNAm was measured using Illumina Infinium HumanMethylation450 BeadChips (Illumina, San Diego, CA) according to the manufacturer’s protocol [82].

During DNAm data collection, repeats and randomization of samples across chips and plates were used to reduce the chance for artifactual confounding [74]. Quality control of DNAm data excluded samples with poor data quality, i.e., poor median methylated and unmethylated signal levels and with 1% of probes fluorescing below the limit of detection (*n *= 0); mismatch between recorded sex and sex estimated from DNAm data (*n *= 8), and replicate samples (*n *= 1). Functional normalization [83] was performed using the R package *minfi* (v1.36.0) [84] and normalizing of signal levels of type I and type II probes performed using the package *ENmix* (v1.26.10) [85]. The ComBat [86] method provided in the *sva* package (v3.38.0) [87] was used to remove batch effects associated with the bisulfite conversion step. Proportions of seven cell types (CD8 + T cells, CD4 + T cells, natural killer cells, B cells, monocytes, granulocytes, and nucleated red blood cells) were estimated from cord blood methylation profiles, using the method of Teschendorff et al. as provided in the package *EpiDISH* (v2.6.1) [88].

### Statistical analysis

*Epigenome-wide association analysis (EWAS), differentially methylated positions (DMPs).* A total of 372 samples were retained with high-quality DNAm data, of which 196 with complete paired data on maternal ACEs and covariates were used for EWAS analyses. We performed three complementary EWAS that differed in the definition of maternal ACE exposures: (1) we applied the simplifying assumption that exposure to additional types of adverse experience has homogeneous effects on DNAm, treating the total number of ACEs as a continuous variable modeled linearly; (2) we regarded each additional type of adverse experience as homogenous and categorized the total number of ACEs as 0, 1–3, or 4–10 ACEs, allowing for nonlinear effects; and (3) we regarded each of the 10 indicators for different ACEs as distinct exposures, fitting coefficients for each in a mutually adjusted model. For each exposure definition, we fit linear models on the logit scale (*M*-values) with empirical Bayes adjustment of standard error terms using the package *limma* (v3.46.0) [89]. In addition to the exposure terms, models included the following covariates selected *a priori* based on previously reported associations with cord blood DNAm [65–74]: newborn sex, gestational age, and estimated nucleated cell-type composition, and maternal parity, pre-pregnancy BMI, age at delivery, educational attainment (6th grade or lower, 7–12th grade, or high school graduate), smoking during pregnancy (ever or never), and marital status at time of birth (married, living as married, separated, divorced, or single having never married). For each CpG, we extracted *p* values, coefficients, and standard errors associated with ACE exposure terms.

While regressing on the logit scale has appealing statistical properties for proportional outcomes [90], the fitted coefficients of a model on this scale describe methylation odds ratios, whereas it is often of interest to know the expected difference in percent methylation associated with an exposure. To this end, we retrieved plug-in estimates of predicted baseline methylation levels by defining a ‘reference individual’ as a child having the mean value of all numeric covariates and the modal value of categorical covariates (sex: female; gestational age: 273.36 days; cell type composition: 18.94% CD4 + T cells, 9.12% CD8 + T cells, 0.73% natural killer cells, 18.33% B Cells, 10.92% monocytes, 40.63% granulocytes, 1.33% nucleated red blood cells; maternal parity: 1.19 births; maternal pre-pregnancy BMI: 27.20; maternal age at delivery: 25.73 years; maternal educational attainment: 6th grade or lower; maternal smoking during pregnancy: no; maternal marital status: married), then contrasted predicted methylation levels for reference individuals with and without the exposure of interest. Confidence intervals for predicted differences in methylation were constructed by Monte Carlo simulating differences in predicted values between exposed and unexposed reference individuals 3000 times as follows:

$$\delta =\frac{1}{1+{e}^{-M\left({\varvec{\theta}},\boldsymbol{ }{{\varvec{X}}}_{1}\right)}}-\frac{1}{1+{e}^{-M\left({\varvec{\theta}},\boldsymbol{ }{{\varvec{X}}}_{0}\right)}}$$

$$M\left({\varvec{\theta}},{\varvec{X}}1\right)=\boldsymbol{\alpha }{\varvec{\theta}}+{\varvec{\beta}}{{\varvec{X}}}_{1}$$

$$M\left({\varvec{\theta}}, {{\varvec{X}}}_{0}\right)=\boldsymbol{\alpha }{\varvec{\theta}}+{\varvec{\beta}}{{\varvec{X}}}_{0}$$

$$\left\{\boldsymbol{\alpha },{\varvec{\beta}}\right\} \sim Multivariate\,normal\left(\left\{\widehat{\boldsymbol{\alpha }},\widehat{{\varvec{\beta}}}\right\}, \widehat{{\varvec{\Sigma}}}\right)$$

where $\delta$ denotes the difference in methylation associated with exposure matrix ${{\varvec{X}}}_{1}$ in contrast to reference matrix ${{\varvec{X}}}_{0}$, ${\varvec{\theta}}$ is a matrix of covariates fixed to their average/modal values, $\boldsymbol{\alpha }$ is a vector of randomly generated coefficients associated with covariates ${\varvec{\theta}}$, ${\varvec{\beta}}$ is a vector of randomly generated coefficients associated with exposures of interest, $\widehat{\boldsymbol{\alpha }}$ and $\widehat{{\varvec{\beta}}}$ are the fitted point estimates of the coefficients, and $\widehat{{\varvec{\Sigma}}}$ is their variance–covariance matrix.

We assessed the distribution of *p* values from each CpG-by-CpG EWAS via quantile–quantile plots and calculated genomic inflation factors ($\lambda$) for each exposure in each model. We adjusted for multiple comparisons, calculating *q* values using the FDR control approach of Storey and Tibshirani [34], as implemented in the R package *q value* (v2.22.0), noting any CpGs as differentially methylated for which a *q* value ≤ 0.05 was returned. Adjustment for multiple comparisons was performed separately for each specification of ACE exposures (i.e., three sets of multiple comparisons adjustments): total number of ACEs as a continuous variable, total number of ACEs as a categorical variable, and individual ACEs.

We conducted a look-up of the one DMP using the EWAS Catalog [35]. We also compared our EWAS results to findings reported by two recent studies. In the first study, associations between the number of maternal ACEs and infant DNAm measured in blood (*N *= 92) were analyzed using correlated methylated regions and individual CpGs not included in the regions [29]. The top CpG located in each 320 regions (*p* < 0.005) and 189 individual CpGs (*p* < 0.0005) were reported, none of which remained significant after an FDR correction. Although this study also investigated associations with infant DNAm measured in buccal epithelial cells, we limited our look-up to results from blood for greater similarity in tissue types. In the second study, associations of maternal stressful life events experienced during pregnancy and offspring DNAm were studied in a meta-analysis of 12 cohorts (*N *= 5496) conducted by the PACE consortium [26]. Prenatal stressors were analyzed as the cohort-specific proportion of stressors reported or five domains of stressors harmonized across cohorts, and five DMPs were identified (*p* < 2.4 × 10^–7^), four of which were included in our study. To assess if CpGs associated with maternal ACEs or prenatal stress were also associated with ACEs in our study, we conducted a look-up of reported CpGs in our EWAS results. Replication of was determined by a nominal *p* value < 0.05.

*Differentially methylated region (DMR) analysis.* We searched for DMRs using a modified comb-p method as provided in *Enmix* (v1.26.10) [91], with maximum distance between base pairs within a DMR set to 1000 and a seed value (FDR significance threshold for initial selection of DMR regions) of 10^–3^ [38]. We defined significant DMRs as those with ≥ 3 CpGs and a Šidák-adjusted *p* value ≤ 0.05.

*Weighted gene co-expression network analysis (WGCNA).* We utilized the R package *WGCNA* (v1.70.3) [39] to perform weighted correlation network analysis, or weighted gene co‐expression network analysis (WGCNA), of methylation *M-*values across the epigenome. *WGCNA* provides a suite of tools for tasks including the identification of non-contiguous network modules of covarying signal loci, identification of central loci in the network (e.g., clusters of covarying CpG probes situated on different chromosomes), and extraction of summary statistics of module-wide signals such as the *eigengene* [92] (a measure of distance along the module’s principal axis of variation) which can then be related to exposures or traits of interest. We used the *blockwiseModules* function to feasibly calculate unsigned Topological Overlap Matrices for our high dimensional methylation data, then identify covarying modules via hierarchical clustering and dynamic tree cutting algorithms [93] and calculated module eigengenes (MEs) for each subject. One module was composed primarily of probes located on the sex chromosomes and was excluded from further analyses.

To identify modules associated with maternal ACEs, we calculated Pearson correlations between MEs and total ACEs, as well as individual ACEs separately. Analogous to our approach for FDR control in our EWAS analysis, we estimated *q* values separately for each specification of ACE exposures: total number of ACEs as a continuous variable, total number of ACEs as a categorical variable, and individual ACEs. For linear and categorical specifications of total ACEs (28 and 56 tests, respectively), we used the Benjamini–Hochberg FDR estimation, which has higher specificity at low sample sizes but reduced power [94, 95]. For individual ACEs (280 tests), we used the estimation Strimmer method for estimating *q* values [94, 96]. For ME-ACE relationships exhibiting a correlation *p* value ≤ 0.05, we assessed the robustness of the association by constructing linear regressions with the ME as outcome, ACE measure as main exposure, and adjusting for newborn sex, gestational age, and estimated nucleated cell-type composition, and maternal parity, pre-pregnancy BMI, age at delivery, educational attainment, smoking during pregnancy, and marital status. For individual ACEs, we additionally assessed the significance of their association with MEs in a mutually adjusted model in which all ACEs were included simultaneously along with the covariates listed above. FDRs were estimated only for bivariate associations to provide an indication of the level of confidence of the probability that associations in our exploratory analyses did not arise by change under multiple testing. FDR estimates were not calculated for adjusted modules, since these estimates had already been subjected to a selection process informed by the same data.

We performed KEGG [40] and GO [41, 42] pathway enrichment analyses for modules significantly associated with ACE exposures after adjustment for other covariates using the *gometh* function in the *missMethyl* package (v1.26.1). This function accepts a vector of CpGs included in each module and a vector of all CpGs used in WGCNA. Enrichment *p* values were adjusted for prior gene selection probabilities inherent to the Illumina array [97, 98], and pathways with intra-module FDR ≤ 0.05 were considered to be enriched.

### Supplementary Information

**Additional file 1: Table S1**. Participant characteristics of mother/newborn pairs with cord blood DNA methylation data (*N *= 372). **Table S2**. Weighted correlation analysis (WGCNA) module eigengene representativeness statistics. **Table S3**. Weighted correlation analysis (WGCNA) module eigengenes (MEs) associated with ACE exposures and pathway summaries. **Fig. S1**. Flowchart of selection process for mother/newborn pairs included in analyses. **Fig. S2**. Heatmap of pairwise odds ratios between maternal adverse childhood experiences (ACEs). **Fig. S3**. Manhattan plot for associations with the total number of maternal adverse childhood experiences (ACEs) modeled linearly. **Fig. S4**. Manhattan plots for associations with the total number of maternal adverse childhood experiences (ACEs) categorized as 0, 1–3, or 4–10. **Fig. S5**. Manhattan plots for associations with individual maternal adverse childhood experiences (ACEs) in a mutually adjusted model. **Fig. S6**. Q–Q plot for associations with the total number of maternal adverse childhood experiences (ACEs) modeled linearly. **Fig. S7**. Q–Q plots for associations with the total number of maternal adverse childhood experiences (ACEs) categorized as 0, 1–3, or 4–10. **Fig. S8**. Q–Q plots for associations with individual maternal adverse childhood experiences (ACEs) in a mutually adjusted model. **Fig. S9**. Correlations of module eigengenes (MEs) with individual maternal adverse childhood experience (ACE) indicators, total number of maternal ACEs, and covariates.

**Additional file 2: Table S1**. Lookup of Moore et al. results including top CpG located in each correlated methylated region (*p* < 0.005) and individual CpGs (*p* < 0.0005), and EWAS results from the current study. **Table S2**. Lookup of Kotsakis Ruehlmann et al. results including CpGs associated with prenatal cumulative stress or individual stress domains in PACE (*p* value  <  2.4  ×  10^−7^), and EWAS results from the current study.

**Additional file 3: Table S1.** Kyoto Encyclopedia of Genes and Genomes (KEGG) pathways enriched for modules associated with ACE exposures (FDR < 0.05). **Table S2. **Gene Ontology (GO) pathways enriched for modules associated with ACE exposures (FDR < 0.05).

## Data Availability

The datasets supporting the conclusions of this article are not publicly available; consent for public release of epigenetic data was not obtained from all participants. However, full CpG-by-CpG results from the EWAS and information required to reconstruct identified WGCNA modules are available at the study’s Open Science Framework (OSF) repository (https://osf.io/ync5t/). Additional output to generate figures and tables is available from the corresponding author with the appropriate permission from the CHAMACOS team and investigators upon reasonable request and Institutional Review Board approval.

## Abbreviations

ACES: Adverse childhood experiences
DNAm: DNA methylation
EWAS: Epigenome-wide association study
CHAMACOS: Center for the Health Assessment of Mothers and Children of Salinas
SD: Standard deviation
BMI: Body mass index
CI: Confidence interval
CpG: Cytosine-phosphate-guanine dinucleotide
FDR: False discovery rate
DMP: Differentially methylated position
OSF: Open science framework
PACE: Pregnancy and Childhood Epigenetics Consortium
DMR: Differentially methylated region
WGCNA: Weighted gene co-expression network analysis
ME: Module eigengene
KEGG: Kyoto Encyclopedia of Genes and Genomes
GO: Gene ontology
PTSD: Post-traumatic stress disorder
MHC: Major histocompatibility complex
