# Supplementary material for: Maternal adverse childhood experiences (ACEs) and DNA methylation of newborns in cord blood
Source: Clin Epigenetics. 2023 Oct 16;15:162. doi: 10.1186/s13148-023-01581-y (PMC10577922; doi:10.1186/s13148-023-01581-y)
Supplement: Supplementary file 1 — Additional file 1: Table S1. Participant characteristics of mother/newborn pairs with cord blood DNA methylation data (N = 372). Table S2. Weighted correlation analysis (WGCNA) module eigengene representativeness statistics. Table S3. Weighted correlation analysis (WGCNA) module eigengenes (MEs) associated with ACE exposures and pathway summaries. Fig. S1. Flowchart of selection process for mother/newborn pairs included in analyses. Fig. S2. Heatmap of pairwise odds ratios between maternal adverse childhood experiences (ACEs). Fig. S3. Manhattan plot for associations with the total number of maternal adverse childhood experiences (ACEs) modeled linearly. Fig. S4. Manhattan plots for associations with the total number of maternal adverse childhood experiences (ACEs) categorized as 0, 1–3, or 4–10. Fig. S5. Manhattan plots for associations with individual maternal adverse childhood experiences (ACEs) in a mutually adjusted model. Fig. S6. Q–Q plot for associations with the total number of maternal adverse childhood experiences (ACEs) modeled linearly. Fig. S7. Q–Q plots for associations with the total number of maternal adverse childhood experiences (ACEs) categorized as 0, 1–3, or 4–10. Fig. S8. Q–Q plots for associations with individual maternal adverse childhood experiences (ACEs) in a mutually adjusted model. Fig. S9. Correlations of module eigengenes (MEs) with individual maternal adverse childhood experience (ACE) indicators, total number of maternal ACEs, and covariates. [file 13148_2023_1581_MOESM1_ESM.docx]

**Maternal Adverse Childhood Experiences (ACEs) and DNA methylation of newborns in cord blood**

**Additional File 1**

**Table S1:** Participant characteristics of mother/newborn pairs with cord blood DNA methylation data (N = 372).

| **Characteristic** | **Mean ± SD or N (%)** |
| --- | --- |
| **Maternal age at delivery (years)** | 25.3 **±** 5.0 |
| **Maternal parity (births)** | 1.2 **±** 1.3 |
| **Maternal pre-pregnancy BMI** | 26.8 **±** 5.1 |
| **Highest level of education attained by mother** |  |
| 6^th^ grade or lower | 157 (42.2%) |
| 7-12^th^ grade | 138 (37.1%) |
| High school graduate | 77 (20.7%) |
| **Mother ever smoked during pregnancy** |  |
| Yes | 24 (6.5%) |
| No | 346 (93.0%) |
| Missing | 2 (0.5%) |
| **Mother’s marital status at time of birth** |  |
| Married | 161 (43.3%) |
| Living as married | 132 (35.5%) |
| Separated | 19 (5.1%) |
| Divorced | 3 (0.8%) |
| Single, never married | 55 (14.8%) |
| Missing | 2 (0.5%) |
| **Mother’s country of origin** |  |
| US | 47 (12.6%) |
| Mexico | 318 (85.5%) |
| Other | 7 (1.9%) |
| **Years mother spent in US** |  |
| <=1 years | 75 (20.2%) |
| 2-5 years | 114 (30.6%) |
| 6-10 years | 89 (23.9%) |
| 11+ years | 94 (25.3%) |
| **Newborn sex** |  |
| Male | 183 (49.2%) |
| Female | 189 (50.8%) |
| **Gestational age (weeks)** | 39.0 **±** 1.6 |

**Table S2:** Weighted correlation analysis (WGCNA) module eigengene representativeness statistics.

| **Module (#CpGs)** | **Eigengene ^a^ % *M*-value variance explained** | **Median (95% CI) eigengene probe loading ^b^** |
| --- | --- | --- |
| LightCyan1 (48) | 69.41% | 0.87 (0.54, 0.97) |
| Blue (109,576) | 43.03% | 0.65 (0.36, 0.87) |
| Pink (12,955) | 44.52% | 0.65 (0.44, 0.85) |
| SkyBlue4 (63) | 60.69% | 0.77 (0.64, 0.90) |
| Salmon (2,531) | 47.96% | 0.68 (0.43, 0.90) |
| SaddleBrown (94) | 60.55% | 0.82 (0.53, 0.92) |
| RoyalBlue (296) | 55.71% | 0.75 (0.62, 0.86) |
| Magenta (3,363) | 41.01% | 0.63 (0.45, 0.81) |
| MidnightBlue (822) | 59.56% | 0.79 (0.56, 0.89) |
| Brown (91,675) | 44.21% | 0.68 (0.33, 0.85) |
| Turquoise (84,323) | 49.69% | 0 .72 (0.39, 0.89) |
| Ivory (85) | 57.87% | 0.75 (0.66, 0.87) |
| Sienna4 (33) | 61.18% | 0.72 (0.58, 0.96) |
| DarkOliveGreen (72) | 59.31% | 0.79 (0.63, 0.87) |
| Yellow3 (59) | 54.92% | 0.73 (0.62, 0.91) |
| Violet (71) | 62.30% | 0.78 (0.50, 0.98) |
| MediumPurple4 (73) | 59.94% | 0.77 (0.70, 0.86) |
| LightCyan (576) | 64.68% | 0.81 (0.52, 0.93) |
| Coral3 (276) | 61.38% | 0.78 (0.62, 0.93) |
| Green (15944) | 43.99% | 0.64 (0.36, 0.92) |
| LightYellow (182) | 61.13% | 0.79 (0.48, 0.92) |
| GreenYellow (1919) | 49.58% | 0.69 (0.59, 0.84) |
| Red (8879) | 47.75% | 0.68 (0.51, 0.86) |
| Pink4 (30) | 74.03% | 0.91 (0.52, 0.95) |
| DarkSeaGreen4 (34) | 69.24% | 0.86 (0.60, 0.95) |
| Yellow (2376) | 53.29% | 0.74 (0.43, 0.90) |
| Cyan (879) | 52.15% | 0.72 (0.53, 0.87) |
| LightSteelBlue1 (47) | 66.11% | 0.82 (0.66, 0.92) |
| a. Module eigengenes are constructed from the first principal component of variation of *M*-values across probes assigned to the module. | | |
| b. Probe loadings on each eigengene are estimated as the absolute value of the Pearson correlation of *M*-values with eigengene values across samples. | | |

**Table S3:** Weighted correlation analysis (WGCNA) module eigengenes (MEs) associated with ACE exposures and pathway summaries. Only MEs and ACE exposures with significant correlations (Pearson correlation *p* < 0.05) are shown. Significant *p*-values are bolded.

|  |  | **Bivariate ^a^** | | **Adjusted ^b^** | **Mutually adjusted** | **Pathway analysis summary ^d^** |
| --- | --- | --- | --- | --- | --- | --- |
| **Module (#CpGs)** | **ACE exposure** | ***ρ*** | ***p*-value (*q*-value)** | ***p-*value** | ***p*-value ^c^** |  |
| LightCyan1 (48) | Sexual abuse | -0.14 | **0.046** (0.39) | 0.065 | **0.016** | - |
| Blue (109,576) | Emotional abuse | -0.15 | **0.039** (0.37) | 0.090 | 0.093 | Neurodegenerative disease / cellular anatomy and metabolism |
| Pink (12,955) | Mental illness | -0.16 | **0.022** (0.30) | 0.078 | 0.16 | Cellular metabolic and biosynthetic processes |
| SkyBlue4 (63) | Emotional abuse | -0.15 | **0.039** (0.37) | 0.058 | 0.29 | - |
|  | Physical abuse | -0.16 | **0.026** (0.32) | **0.020** | 019 |  |
|  | Divorce | -0.16 | **0.024** (0.31) | **0.049** | 0.20 |  |
| Salmon (2,531) | Divorce | 0.14 | **0.044** (0.39) | **0.022** | **0.036** | Immune and inflammatory response / cell death and cancer |
| SaddleBrown (94) | Emotional neglect | 0.15 | **0.031** (0.34) | **0.031** | **0.017** | Embryonic development |
| RoyalBlue (296) | Mental illness | 0.20 | **0.006** (0.19) | **0.017** | **0.029** | - |
| Magenta (3,363) | Mental illness | 0.20 | **0.006** (0.19) | **0.043** | 0.14 | Immune and inflammatory response |
| MidnightBlue (822) | Emotional abuse | 0.17 | **0.019** (0.29) | **0.039** | 0.39 | Cell-mediated immune response |
|  | Physical abuse | 0.14 | **0.048** (0.40) | **0.034** | 0.66 |  |
|  | Mental illness | 0.22 | **0.002** (0.13) | **0.003** | **0.015** |  |
|  | Divorce | 0.16 | **0.026** (0.31) | **0.050** | 0.27 |  |
|  | Total number of ACEs | 0.15 | **0.039** (0.36) | **0.042** | **-** |  |
|  | Total number of ACEs categorized | 1-3 vs. 0: 0.04 | 0.62 (0.91) | 0.79 | **-** |  |
|  |  | 4-10 vs. 0: 0.15 | **0.045** (0.66) | **0.044** | **-** |  |
| Brown (91,675) | Emotional abuse | 0.15 | **0.034** (0.35) | **0.045** | 0.074 | ErbB and oxytocin signaling / cell projection |
| Turquoise (84,323) | Mental illness | 0.16 | **0.026** (0.31) | 0.070 | 0.12 | Ion transport and cell signaling |
| Ivory (85) | Domestic violence | 0.18 | **0.012** (0.25) | 0.072 | 0.061 | Cell adhesion and ion binding |
| Sienna4 (33) | Sexual abuse | -0.24 | **<0.001** (0.085) | **<0.001** | **0.012** | - |
|  | Physical neglect | -0.20 | **0.006** (0.20) | **0.007** | 0.27 |  |
|  | Mental illness | -0.18 | **0.010** (0.23) | **0.006** | 0.39 |  |
|  | Total number of ACEs | -0.20 | **0.006** (0.082) | **0.003** | - |  |
|  | Total number of ACEs categorized | 1-3 vs. 0: 0.04 | **0.57** (0.66) | 0.54 | - |  |
|  |  | 4-10 vs. 0: -0.16 | **0.034** (0.61) | **0.023** | - |  |
| DarkOliveGreen (72) | Physical neglect | -0.15 | **0.039** (0.37) | 0.085 | 0.12 | - |
|  | Mental illness | -0.17 | **0.015** (0.27) | **0.021** | 0.07 |  |
| Yellow3 (59) | Sexual abuse | -0.19 | **0.007** (0.20) | **0.013** | 0.53 | - |
|  | Emotional neglect | -0.19 | **0.009** (0.22) | **0.011** | 0.12 |  |
|  | Domestic violence | -0.16 | **0.024** (0.31) | 0.055 | 0.89 |  |
|  | Physical neglect | -0.24 | **<0.001** (0.085) | **0.002** | 0.16 |  |
|  | Substance abuse | -0.16 | **0.022** (0.30) | **0.031** | 0.62 |  |
|  | Mental illness | -0.20 | **0.005** (0.19) | **0.017** | 0.18 |  |
|  | Total number of ACEs | -0.22 | **0.002** (0.51) | **0.006** | - |  |
|  | Total number of ACEs categorized | 1-3 vs. 0: -0.09 | **0.21 (**0.80) | **0.47** | - |  |
|  |  | 4-10 vs. 0: -0.19 | **0.010** (0.61) | **0.025** | - |  |
| a. Bivariate associations were evaluated using Pearson correlations. *q*-values were estimated for bivariate associations grouped by model specifications: *q*-values for individual ACEs were estimated using the *q*-value method introduced by Strimmer [1], whereas q-values for continuous and categorical total ACEs were estimated separately using the Benjamini-Hochberg method [2] due to insufficient sample size for reliable estimation of the overall proportion of null test statistics.  b. Adjusted associations were evaluated using linear models adjusted for newborn sex, gestational age, and cord blood estimated cell type proportions, and maternal parity, pre-pregnancy BMI, age at delivery, educational attainment, smoking during pregnancy and marital status.  c. Mutually adjusted associations were evaluated using adjusted linear models including all covariates and ACE indicators.  d. Summary of pathway enrichment analysis for was also performed for significant modules using the Kyoto Encyclopedia of Genes and Genomes (KEGG) and Gene Ontology (GO) databases. Full pathway analysis results included in Additional File 2 Tables S1 and S2. | | | | | | |

**Figure S1:** Flowchart of selection process for mother/newborn pairs included in analyses. From an initial cohort of 601 mother/newborn pairs, 372 had high-quality cord blood DNA methylation data and were considered for inclusion in the current study.

**
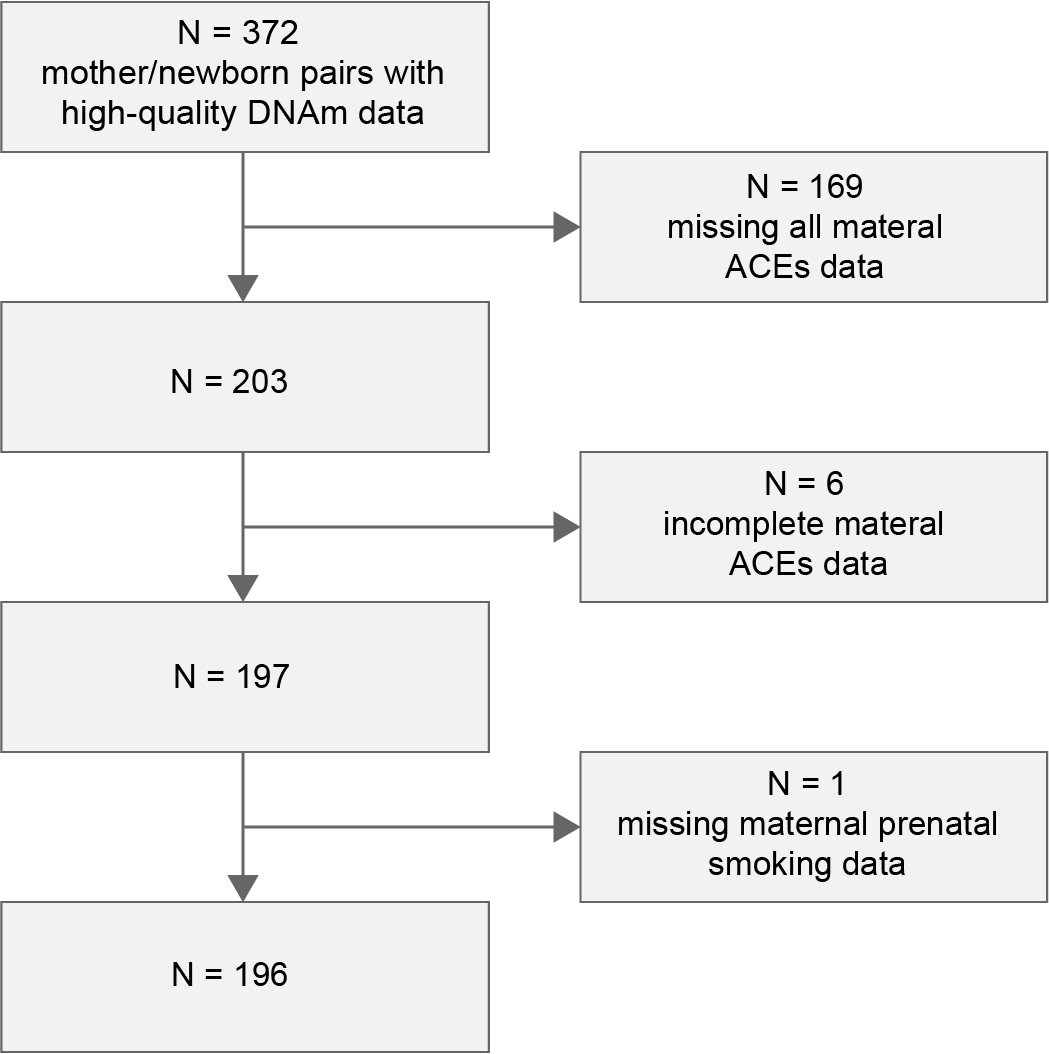
**

**Figure S2:** Heatmap of pairwise odds ratios between maternal adverse childhood experiences (ACEs).


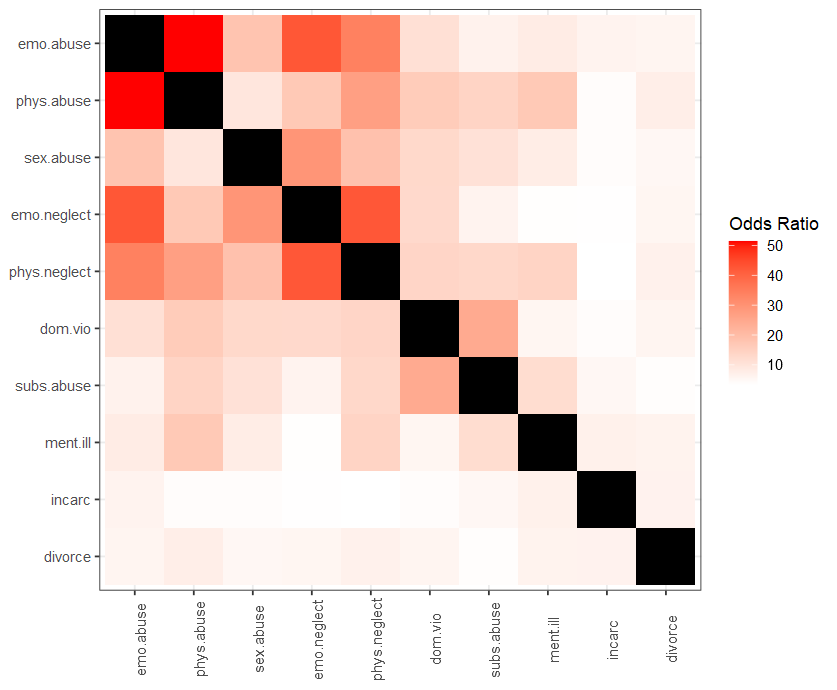


**Figure S3:** Manhattan plot for associations with total number of maternal adverse childhood experiences (ACEs) modeled linearly. Results are from a model adjusted for newborn sex, gestational age, and cord blood estimated cell type proportions, and maternal parity, pre-pregnancy BMI, age at delivery, educational attainment, smoking during pregnancy and marital status.


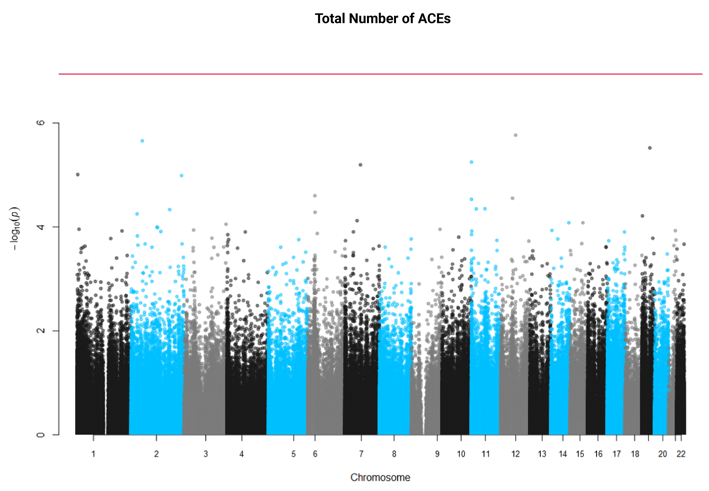


**Figure S4:** Manhattan plots for associations with the total number of maternal adverse childhood experiences (ACEs) categorized as 0, 1-3, or 4-10. Results are from models adjusted for newborn sex, gestational age, and cord blood estimated cell type proportions, and maternal parity, pre-pregnancy BMI, age at delivery, educational attainment, smoking during pregnancy and marital status.


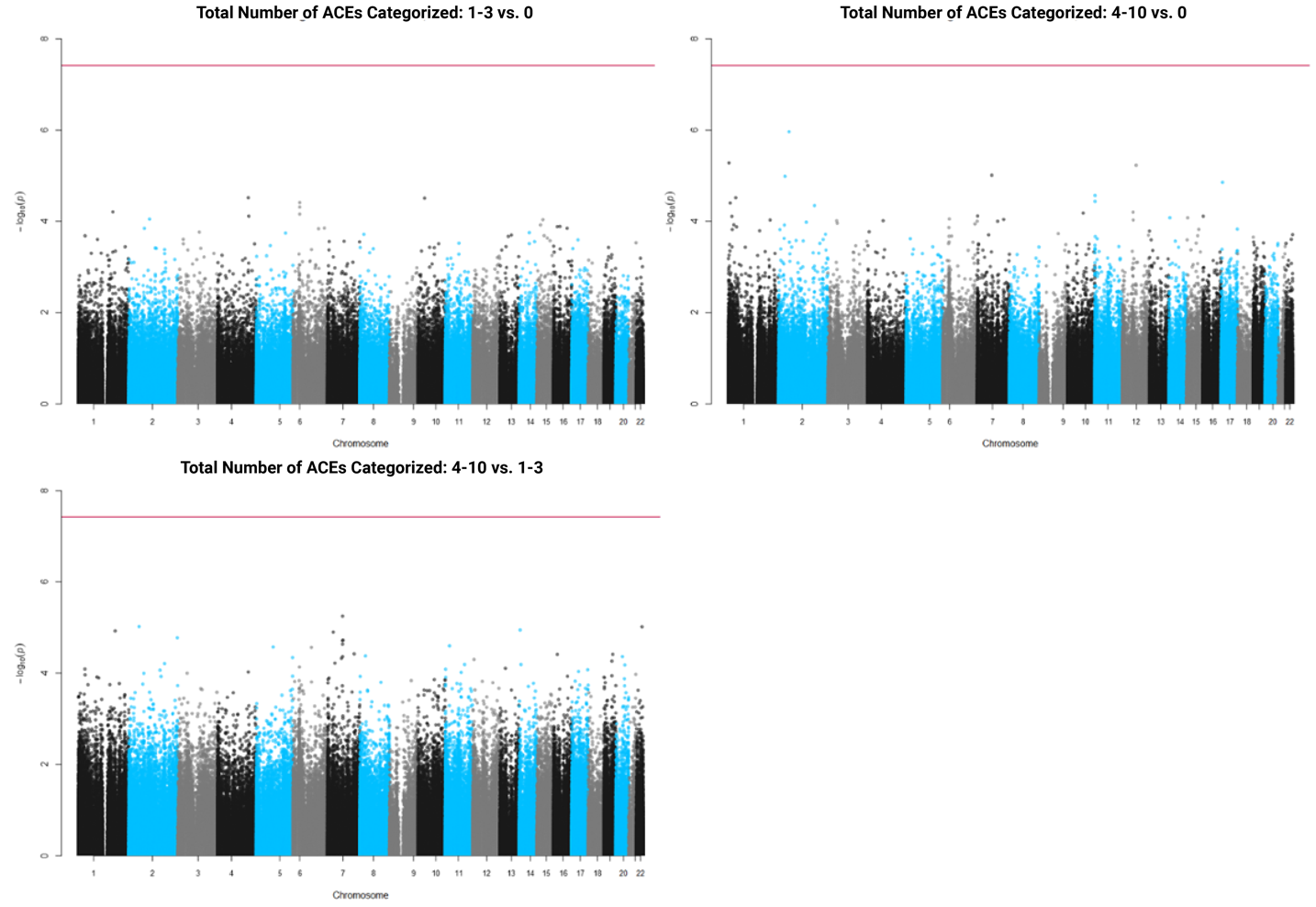


**Figure S5:** Manhattan plots for associations with individual maternal adverse childhood experiences (ACEs) in a mutually adjusted model. Results are from a model adjusted for newborn sex, gestational age, and cord blood estimated cell type proportions, and maternal parity, BMI, age at delivery, educational attainment, smoking during pregnancy and marital status.


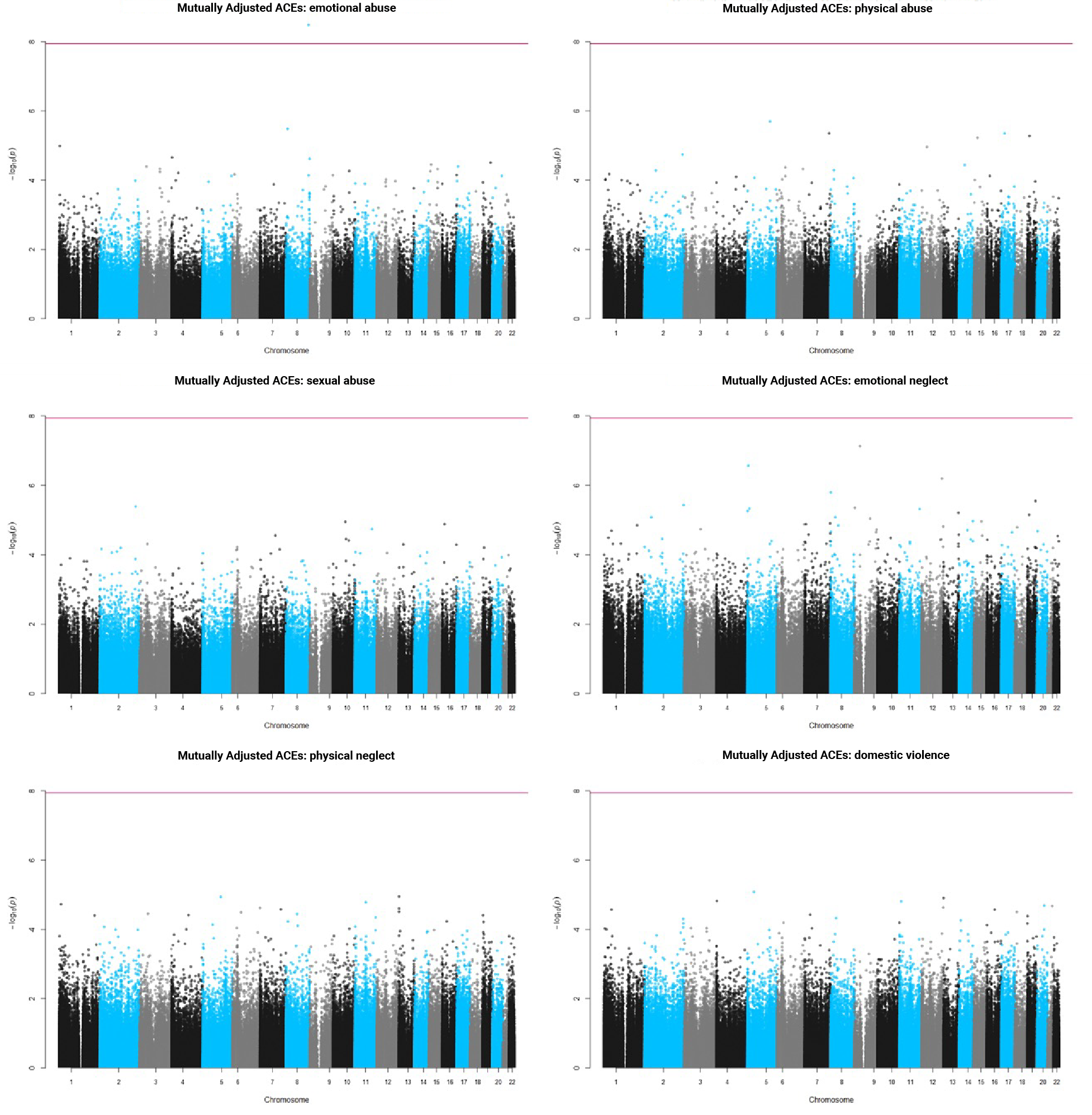


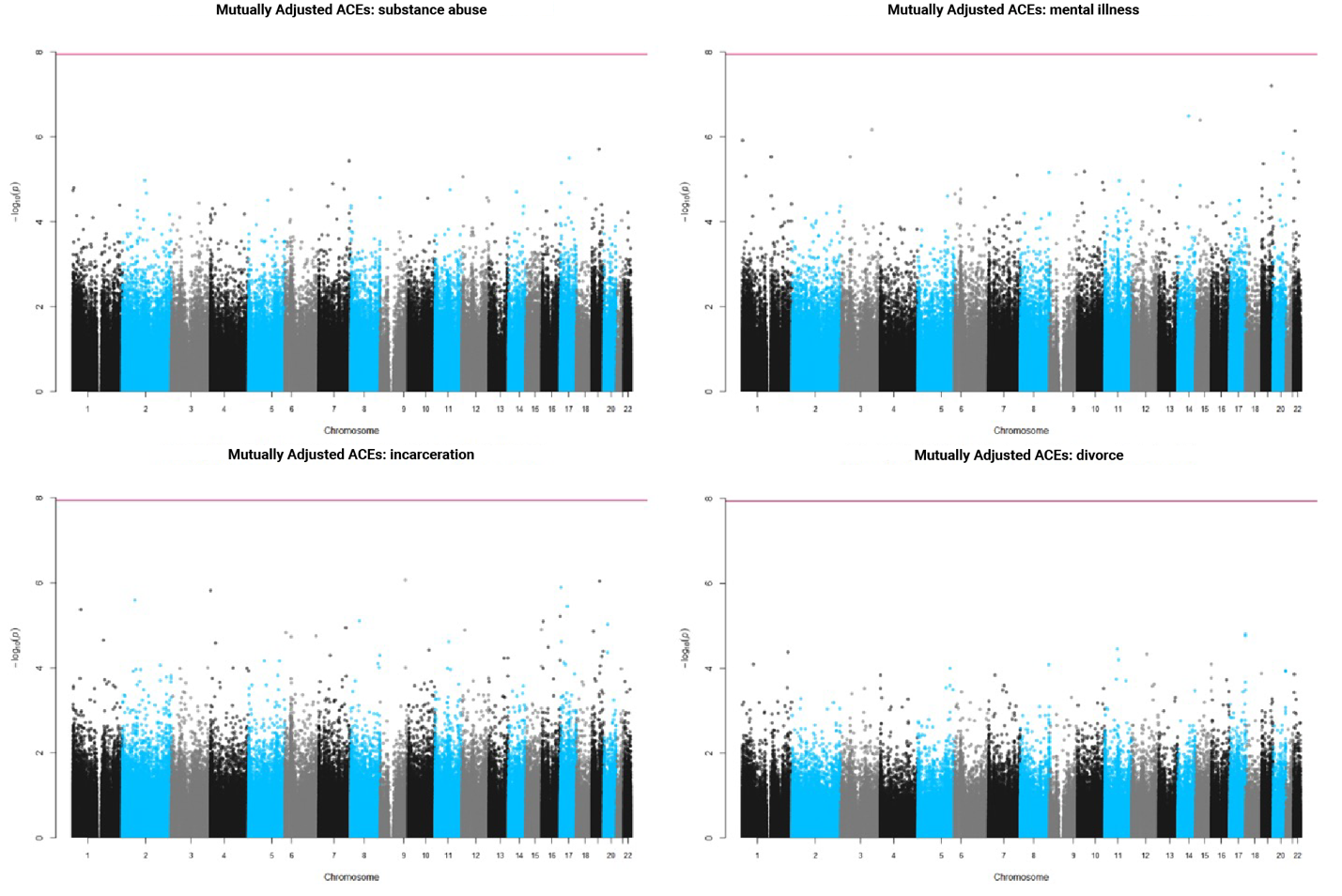


**Figure S6:** Q-Q plot for associations with the total number of maternal adverse childhood experiences (ACEs) modeled linearly. Results are from a model adjusted for newborn sex, gestational age, and cord blood estimated cell type proportions, and maternal parity, pre-pregnancy BMI, age at delivery, educational attainment, smoking during pregnancy and marital status.


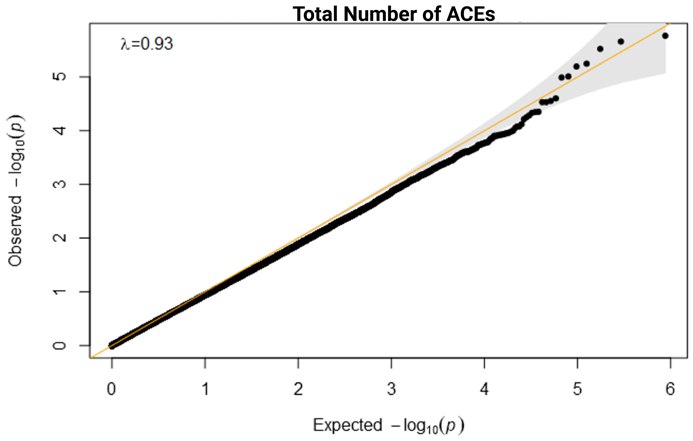


**Figure S7:** Q-Q plots for associations with the total number of maternal adverse childhood experiences (ACEs) categorized as 0, 1-3, or 4-10. Results are from models adjusted for newborn sex, gestational age, and cord blood estimated cell type proportions, and maternal parity, pre-pregnancy BMI, age at delivery, educational attainment, smoking during pregnancy and marital status.


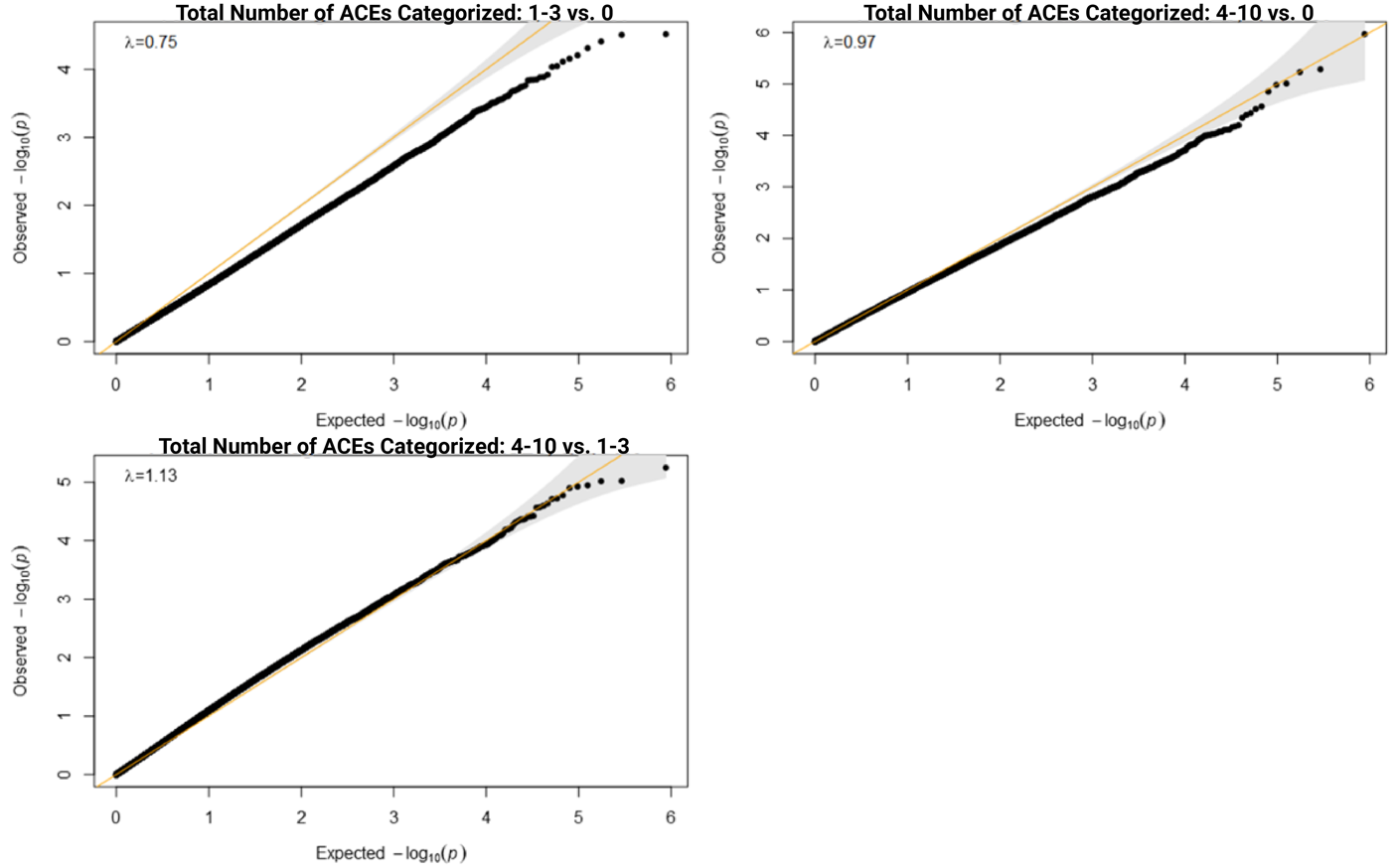


**Figure S8:** Q-Q plots for associations with individual maternal adverse childhood experiences (ACEs) in a mutually adjusted model. Results are from a model adjusted for newborn sex, gestational age, and cord blood estimated cell type proportions, and maternal parity, pre-pregnancy BMI, age at delivery, educational attainment, smoking during pregnancy and marital status.


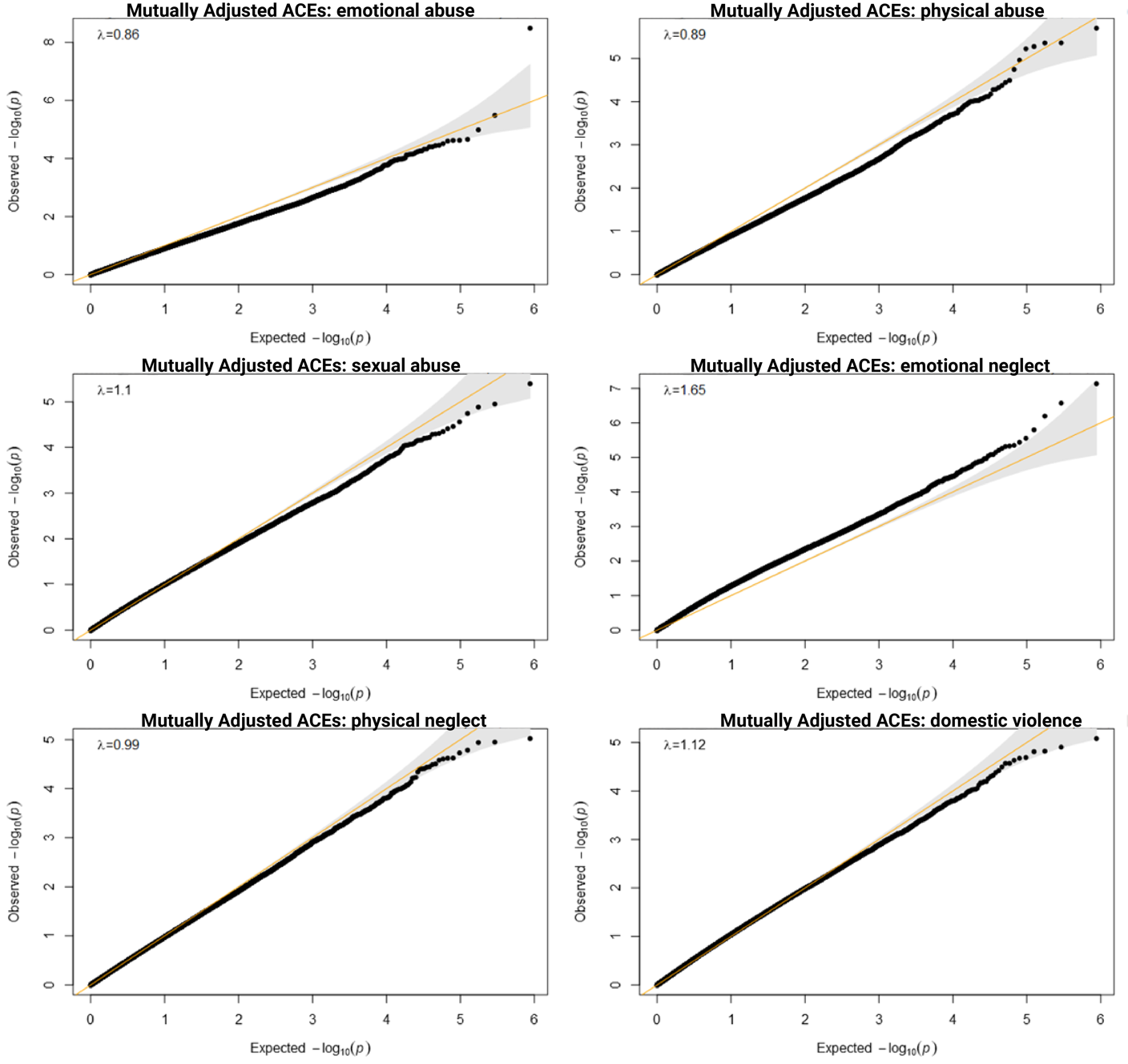


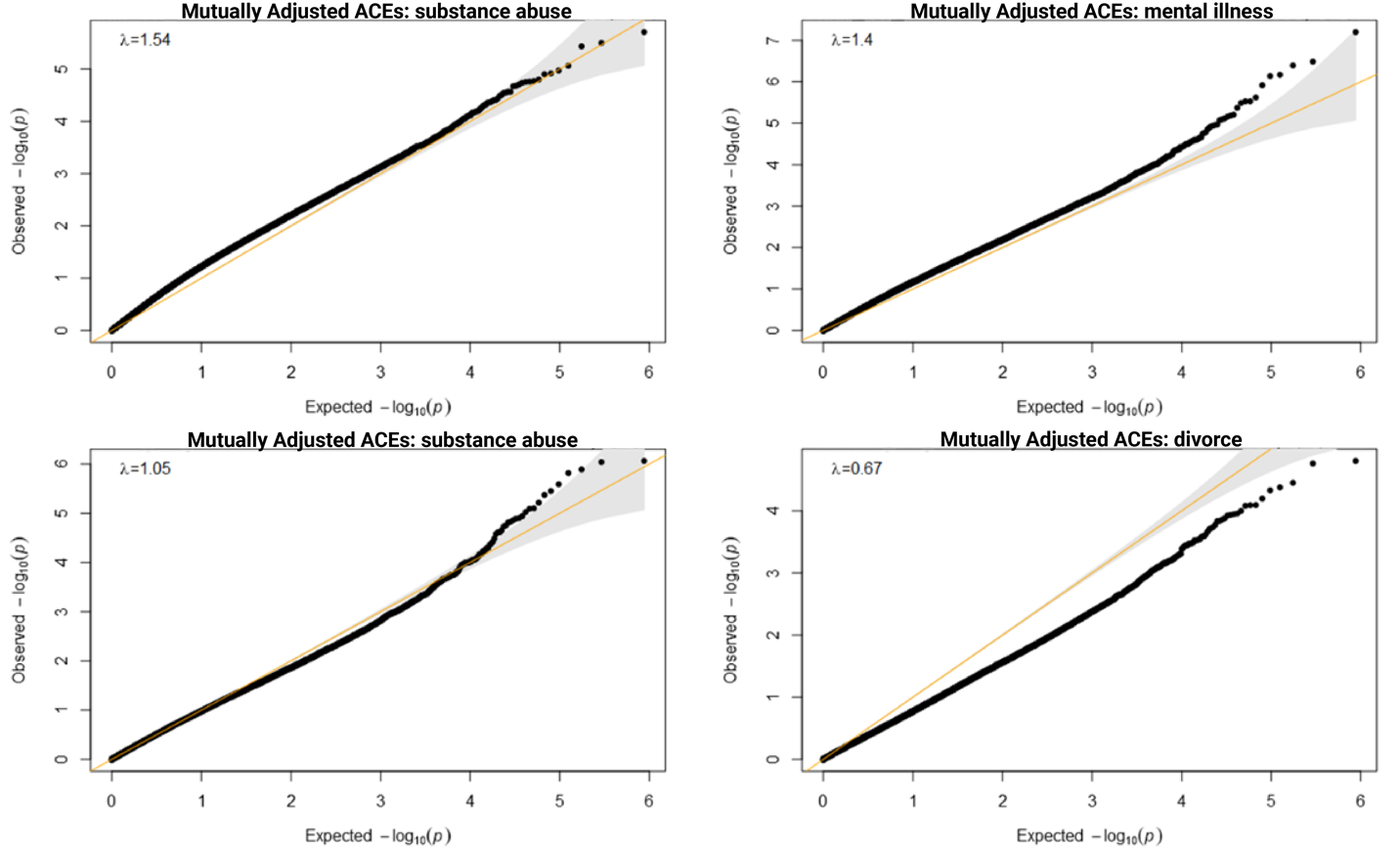


**Figure S9:** Correlations of module eigengenes (MEs) with individual maternal adverse childhood experience (ACE) indicators, total number of maternal ACEs, and covariates. Modules derived from weighted correlation analysis (WGCNA). Pearson correlations (*p*-values; *q*-values) are shown in cells. MEs not significantly correlated with the total number of ACEs or individual ACEs (*p* > 0.05) are shown; MEs significantly correlated with the total number of ACEs or individual ACEs (*p* < 0.05) are shown in the main text **Figure 1**.

**References**

1. Strimmer K. A unified approach to false discovery rate estimation. BMC Bioinformatics. 2008;9:303.

2. Benjamini Y, Hochberg Y. Controlling the false discovery rate: A practical and powerful approach to multiple testing. J R Stat Soc. 1995;57:289–300.
